# Reservoir characterization and rock typing of the gas-bearing El Wastani Formation, Simian Field, offshore Nile Delta

**DOI:** 10.1038/s41598-025-31825-1

**Published:** 2025-12-24

**Authors:** Soliman Anwar, Hassan EL Kadi, Mohamed Reda, Taher Mostafa

**Affiliations:** 1 Rashid Petroleum Company (Shell J.V), New Maadi, Cairo, 11742 Egypt; 2 Faculty of Science, Geology Department, Al-Azhar University, Nasr City, P.O. Box 11884, Cairo, Egypt

**Keywords:** Hydraulic flow units (HFUs), Rock typing, Flow zone indicator (FZI), Nile Delta, El Wastani Formation, Petrophysical analysis, Reservoir characterization, Engineering, Environmental sciences, Hydrology, Solid Earth sciences

## Abstract

Accurate prediction of reservoir performance in heterogeneous, clay-rich clastic systems remains a critical challenge in petrophysics. This study presents a novel, integrated workflow to overcome this challenge by delineating hydraulic flow units (HFUs) for robust rock typing and permeability prediction in the Pliocene El Wastani Formation, Simian field, offshore Nile Delta. Our methodology synergizes advanced petrophysical log analysis, conventional and special core analysis (SCAL), and sedimentological facies characterization to decipher controls on reservoir quality. The multi-technique approach included: spectral gamma ray (Th-K) cross-plots and Thomas-Stieber model analysis to characterize a dominant laminated illite/smectite clay assemblage; MDT pressure data to precisely define the Free Water Level at 2146 m TVDSS; and Pickett plot analysis to determine a formation water resistivity (Rw) of 0.16 Ω.m. Core-based Flow Zone Indicator (FZI) analysis of 208 samples, identified six distinct HFUs, each defined by a unique, high-fidelity porosity–permeability transform (R^2^ = 0.70–0.98). This hydraulic zonation, validated by stratified modified Lorenz (SML) analysis, showed a strong correlation with sedimentary facies, linking high-quality flow units (HFU-4, HFU-5) to high-energy channel deposits. The results quantitatively demonstrate that reservoir quality is primarily governed by the interplay of depositional environment and consequent pore architecture. The superior performance of the RQI/FZI method over the Pittman R35 technique establishes it as the preferred predictive model. This integrated workflow provides a transformative framework for characterizing heterogeneous reservoirs, ultimately enabling optimized well placement, enhanced recovery, and improved reservoir management decisions in the Nile Delta and analogous basins worldwide.

## Introduction

The accurate characterization of subsurface hydrocarbon reservoirs is fundamental to optimizing exploration and production strategies. This process necessitates the integration of petrophysical, sedimentological, and petrographic analyses to decipher critical properties such as pore architecture, fluid saturation, and storage capacity, which collectively define reservoir quality and performance^1–3^. In complex clastic systems, particularly clay-rich gas-bearing formations, reservoir heterogeneity presents a formidable challenge, requiring advanced methodologies to delineate productive zones and mitigate operational risks^2,4^.

**Fig. 1:**
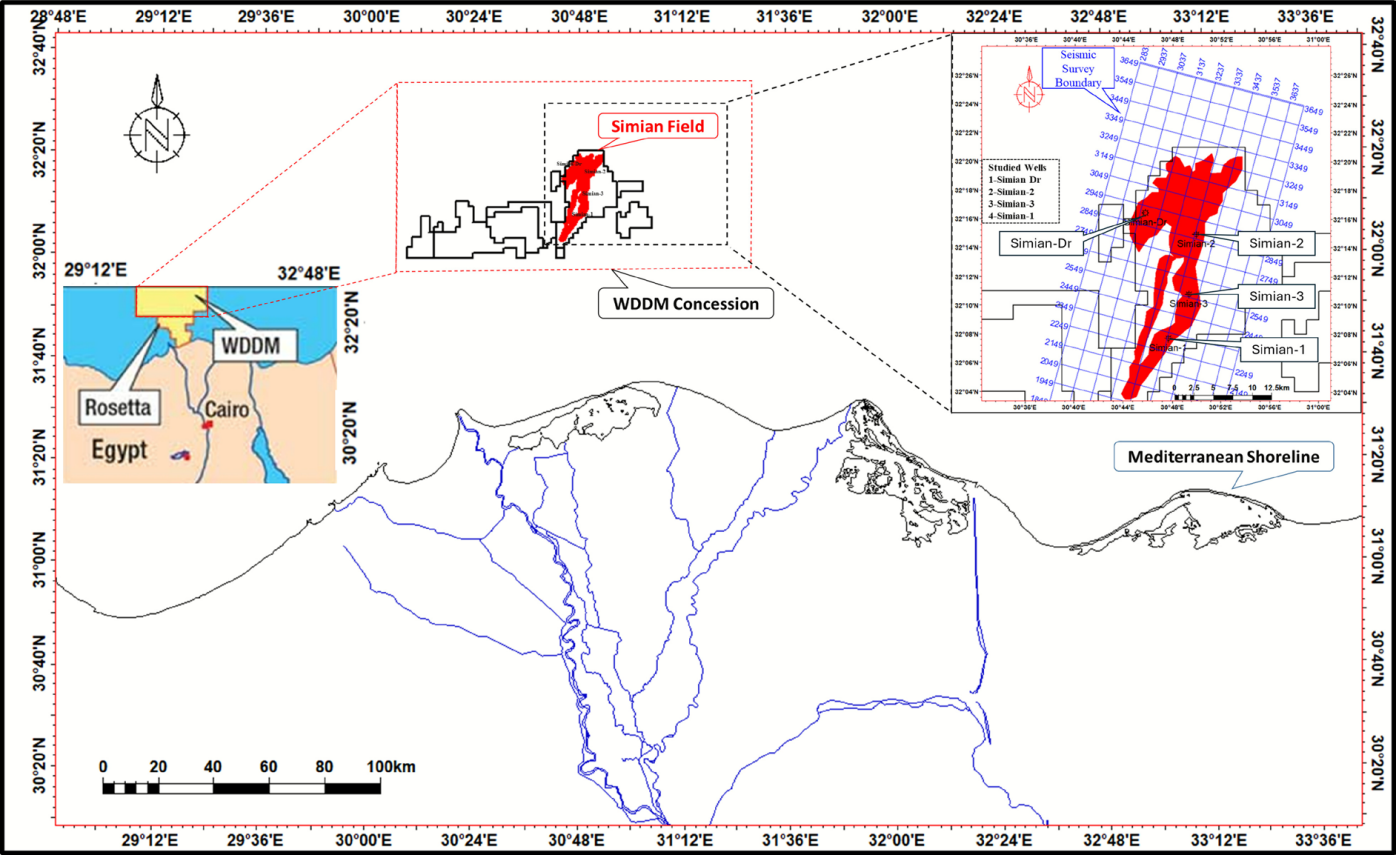
Location map of the simian field within the west delta deep marine (WDDM) concession, offshore Nile Delta, Egypt modified after^6^. The map shows the positions of the four key wells (Simian-1, Simian-2, Simian-3, and Simian-Dr) used in this study.

This study focuses on the Pliocene-aged El Wastani Formation within the Simian gas field, a significant dry-gas accumulation situated in the West Delta Deep Marine (WDDM) concession, offshore Nile Delta, Egypt (Fig. 1). Located approximately 120 km northeast of Alexandria in water depths of 500–1500 m^5,6^, the field is stratigraphically positioned at the transition between the shelfal mudstones of the Kafr El Sheikh Formation and the overlying coastal to fluvio-marine sands of the Mit Ghamr Formation^7^. The El Wastani Formation is characterized by an intercalation of thick sand units and thin clay layers, deposited during the late Pliocene regression that concluded the region’s major sedimentary cycle.

Despite its economic significance, a critical knowledge gap persists in the Nile Delta regarding the synthesis of multi-scale datasets for robust reservoir quality prediction. Previous studies have often relied on seismic interpretation or conventional petrophysical workflows, lacking the granularity to classify rock types and their consequent control on dynamic fluid flow behavior^8^. To address this, we introduce a novel, integrated framework that synergizes comprehensive petrophysical with core data analysis. Our methodology employs advanced techniques, including the Thomas-Stieber model for quantifying clay distribution and multi-parameter cross-plot analysis (e.g., Th-K, Density-Neutron), to overcome the challenges posed by highly heterogeneous and shaly lithologies.

This study integrates petrophysical log interpretation for four wells (Simian-1, Simian-2, Simian-3, and Simian-Dr) with core-derived mineralogical and textural analyses (XRD, SEM) obtained from two conventional cores in the Simian-1 and Simian-2 wells, comprising a total of 208 core plugs (95 from Simian-1 and 113 from Simian-2), to provide a comprehensive rock-typing and hydraulic flow unit characterization. Additionally, pressure data points and a formation water sample from the Simian-1 well were incorporated to enhance the understanding of the reservoir’s fluid distribution and pressure regime. This integrated workflow offers a more detailed and quantitative assessment to capture the reservoir heterogeneity of the slope marine turbidite reservoir compared with previous studies on the El Wastani Formation. By fusing well log data (Gamma Ray, Resistivity, Neutron-Density) with core-derived measurements—including Routine Core Analysis (RCAL) of porosity and permeability, and mineralogical data from XRD and SEM—this study aims to: (1) classify reservoir rocks into distinct petrophysical categories based on their lithology and pore geometry; (2) establish a predictive model for identifying high-quality gas-bearing sands; and (3) develop a calibrated rock-typing framework that provides the foundational workflow for field-wide spatial mapping of reservoir heterogeneity.

## Geologic setting

The Simian Field encompasses an area of approximately 200 km^2^ within the prolific offshore Nile Delta basin. The primary reservoir consists of a Pliocene deep-water turbidite complex, characterized by sandstones and sands of marine origin with heterogeneous reservoir quality^9^. These sediments were sourced from the sandy claystone of the sealing El Wastani Formation and were emplaced as a north/northeast-south/southwest trending slope-channel system draped along the western margin of the Nile Delta slope^10^.

Structurally, the field is situated on the hanging wall of the major Rosetta Fault system. This position implies a combined stratigraphic-structural trapping mechanism, where the architectural style of the turbidite channels is likely influenced by syn-depositional fault activity^11^.

Hydrocarbon generation in the broader Nile Delta province is facilitated by source rocks ranging in age from Mesozoic to Cenozoic. These source facies are highly varied, encompassing lacustrine, terrigenous deltaic, and deep marine deposits, typically associated with periods of relative sea-level rise^12,13^. This diversity in source rock composition and depositional environment has resulted in the accumulation of various hydrocarbon mixtures within the basin, including biogenic gas, sulfur-rich waxy oil, and asphaltenes^14^.

**Fig. 2:**
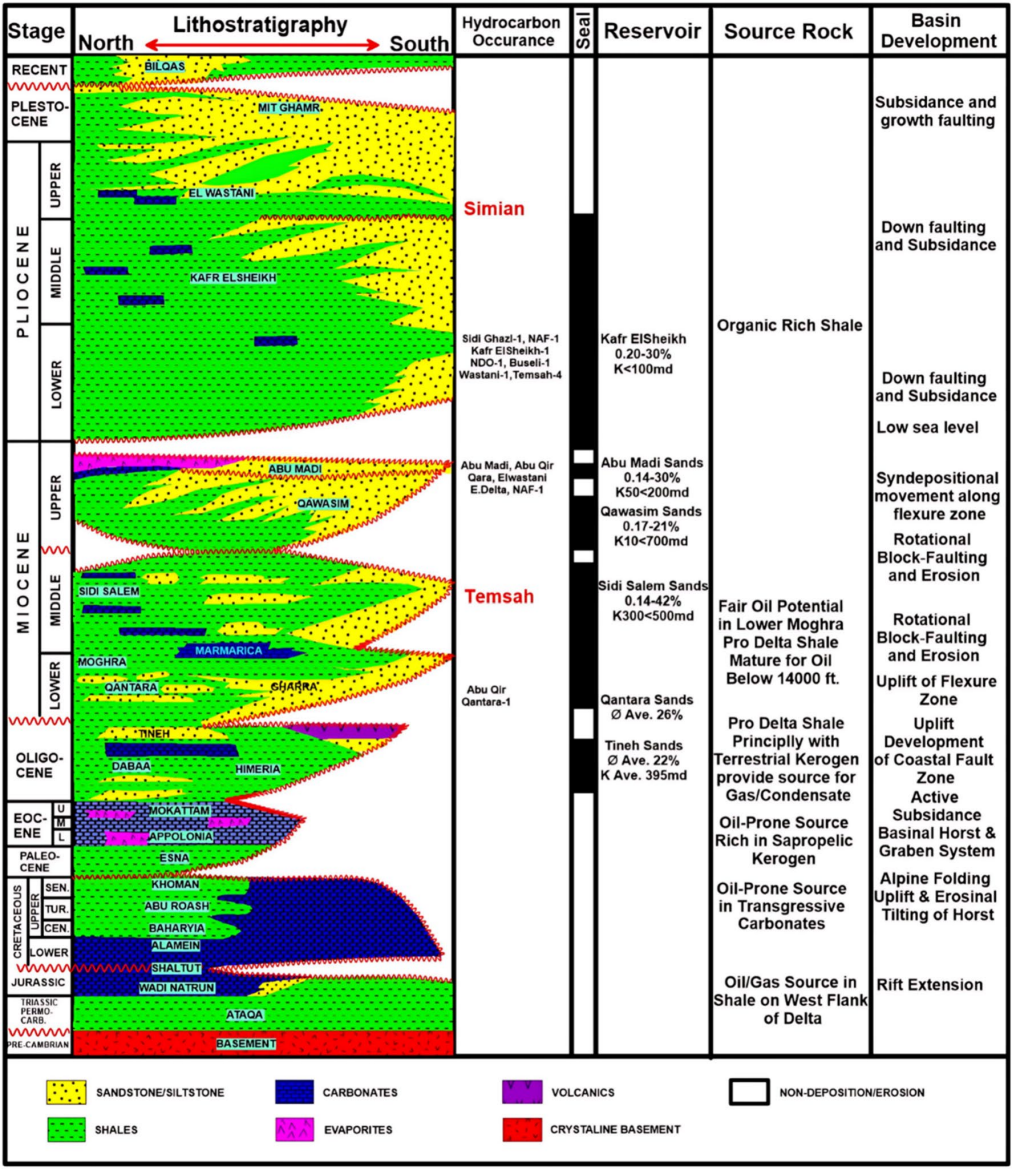
Stratigraphic column of the Simian Field, illustrating the succession from the Bilqas Formation to the El Wastani Formation^6^. The column highlights the primary reservoir interval within the Pliocene-aged El Wastani Formation and its position relative to regional seal and source rocks.

The stratigraphic succession in the Simian Field includes, from oldest to youngest, the Bilqas, Mit Ghamr, and El Wastani formations (Fig. 2). The primary reservoir is hosted within the Pliocene-aged El Wastani Formation, which is interpreted as a complex of slope channels^15^. Reservoir architecture exhibits a clear morphological trend from south to north. The southern segment is characterized by two distinct channel complexes: a larger main channel to the east and a smaller central channel to the west, separated by non-reservoir shale-dominated intervals^16^. These channels are composed of highly sinuous, amalgamated sands with associated levees and frontal-splay deposits. Northward, the channels converge, resulting in decreased confinement and a more bifurcated network of sinuous channel sands.

## Methodology

This study employed an integrated approach, combining well logging data, conventional core analysis, and advanced rock typing techniques to evaluate the reservoir intervals of the Simian gas field. The methodology is divided into three main components: (1) Well Logging Data Analysis, (2) Core Data Analysis, and (3) Rock Typing.

### Well logging data analysis

Digital well logs from four key wells (Simian-1, Simian-2, Simian-3, and Simian-Dr; see Fig. 1) formed the primary dataset for the initial formation evaluation. Prior to any interpretation, a rigorous data pre-processing workflow was applied. This involved depth-matching all log curves to a common reference to ensure depth alignment between different logging runs and tools. Furthermore, comprehensive environmental corrections were performed to account for effects such as borehole washout, mud invasion, and tool standoff, thereby transforming the raw log measurements into data that accurately represent the intrinsic properties of the formation. The analysis has been done using Schlumberger TechLog software.

### Determination of formation temperature (FT)

The accurate determination of in-situ formation temperature is a critical prerequisite for robust petrophysical analysis. Many key reservoir properties exhibit significant temperature dependence, and neglecting to correct for thermal disturbances induced by drilling operations introduces substantial systematic error into subsequent calculations^17^.

$${\text{FT}} = {\text{ST}} + [\left( {\left( {{\text{BHT}} - {\text{ST}}/{\text{TD}}} \right)} \right]*{\text{FD}}$$

where (FT) is formation temperature, (ST) is surface temperature (BHT) is bottom hole temperature, (TD) is total depth and (FD) Formation depth (m).

Formation temperature data were utilized in this study for multiple purposes:

(i) Correction of formation-water resistivity (Rw): Since Rw strongly depends on water salinity, which varies significantly with depth, obtaining an accurate temperature profile from surface to reservoir depth is essential. The temperature log was therefore used to calculate in-situ Rw values using the Arps relation. The corrected Rw values were then incorporated into the Archie equation for water saturation estimation.

(ii) Temperature-dependent corrections to density and porosity in gas-bearing zones: Gas density decreases with increasing temperature, leading to overestimation of apparent porosity derived from the density log. Applying temperature-dependent gas-density corrections minimizes this effect, particularly in high-temperature intervals of the Simian reservoir, ensuring more accurate porosity and saturation evaluations.

#### Lithological identification

Primary lithology was determined using a combination of log curves and cross-plots. The Gamma Ray (GR) log was the primary indicator for distinguishing clean sandstone (low GR) from shaly intervals (high GR). Neutron-Density cross-plots were employed to identify mineral composition and the presence of gas. In sandstone reservoirs, the combination of low-density and low-neutron porosity readings indicates a clean, gas-bearing zone, causing a characteristic crossover effect^18–20^. The M–N plot^21^, a more robust technique for complex mineralogies, was used as a secondary lithology indicator. The M and N parameters were calculated as follows:

$${\varvec{M}}\boldsymbol{ }=\frac{{\varvec{\Delta}}{{\varvec{t}}}_{{\varvec{f}}{\varvec{l}}}-\boldsymbol{ }{\varvec{\Delta}}{\varvec{t}}}{{{\varvec{\rho}}}_{{\varvec{b}}}-\boldsymbol{ }{{\varvec{\rho}}}_{{\varvec{f}}{\varvec{l}}}}\times 0.01$$

$${\varvec{N}}\boldsymbol{ }=\frac{{{\varvec{\phi}}}_{{\varvec{N}}{\varvec{f}}{\varvec{l}}}-\boldsymbol{ }{{\varvec{\phi}}}_{{\varvec{N}}}}{{{\varvec{\rho}}}_{{\varvec{b}}}-\boldsymbol{ }{{\varvec{\rho}}}_{{\varvec{f}}{\varvec{l}}}}$$

where ${\varvec{\Delta}}{\varvec{t}}$ is sonic transit time (µs/ft), ${{\varvec{\rho}}}_{{\varvec{b}}}$ is bulk density (g/cm^3^), ${{\varvec{\phi}}}_{{\varvec{N}}}$ is neutron porosity (v/v), and the subscript fl denotes the value for the interstitial fluid.

The key input parameters used for ***M–N*** calculations are summarized in Table 1. The interstitial fluid values were assumed based on fluid properties at formation temperature ($\boldsymbol{\Delta }{{\varvec{t}}}_{{\varvec{f}}{\varvec{l}}}$ = 189 µs/ft, ${{\varvec{\rho}}}_{{\varvec{f}}{\varvec{l}}}$ = 1.02–1.06 g/cm^3^, ${{\varvec{\phi}}}_{{\varvec{N}}{\varvec{f}}{\varvec{l}}}$ = 1.0 v/v), while the log-derived bulk density (${{\varvec{\rho}}}_{{\varvec{b}}}$), neutron porosity (${{\varvec{\phi}}}_{{\varvec{N}}}$), and sonic transit time (***Δt***) were extracted directly from the logs at representative depth intervals in each well. These parameters were used to compute the ***M*** and ***N*** values employed for lithological discrimination.

**Table 1:** Key input parameters used for the calculation of M and N values for lithological identification in the Simian wells.

Wells	${\varvec{\Delta}}{{\varvec{t}}}_{{\varvec{f}}{\varvec{l}}}$ ${\varvec{\upmu}}\mathbf{s}/\mathbf{f}\mathbf{t}$	${{\varvec{\phi}}}_{{\varvec{N}}{\varvec{f}}{\varvec{l}}}$ **v/v**	${{\varvec{\rho}}}_{{\varvec{f}}{\varvec{l}}}$ g/cc	$${{\varvec{\rho}}}_{\begin{array}{c}b\end{array}}$$ g/cc	${\varvec{\Delta}}\mathbf{t}$ ${\varvec{\upmu}}\mathbf{s}/\mathbf{f}\mathbf{t}$
Simian-1	189	1	1.03	Variable from log readings at different depths	Variable from log readings at each different depths
Simian-2	189	1	1.05		
Simian-3	189	1	1.02		
Simian-Dr	189	1	1.06		

Two conventional cores from the Simian-1 and Simian-2 wells were analyzed by XRD and SEM, and the log-derived lithologies from neutron–density and M–N cross-plots were visually matched to core observations after proper depth correlation.

#### Shale volume estimation

The volume of shale (Vsh) was calculated using the Gamma Ray log. The linear equation was first applied, followed by the non-linear Clavier equation^22,23^ for improved accuracy in formations with high radioactive anomalies. The Clavier equation is given by:

$${{\varvec{V}}}_{{\varvec{s}}{\varvec{h}}}=\boldsymbol{ }1.7\boldsymbol{ }-\boldsymbol{ }\sqrt{3.38\boldsymbol{ }-\boldsymbol{ }{\left({\varvec{I}}{\varvec{G}}{\varvec{R}}\boldsymbol{ }+\boldsymbol{ }0.7\right)}^{2}}$$

where IGR is the Gamma Ray Index, calculated as:

$${\varvec{I}}{\varvec{G}}{\varvec{R}}\boldsymbol{ }=\frac{{\varvec{G}}{{\varvec{R}}}_{{\varvec{l}}{\varvec{o}}{\varvec{g}}}-\boldsymbol{ }{\varvec{G}}{{\varvec{R}}}_{{\varvec{m}}{\varvec{i}}{\varvec{n}}}}{{\varvec{G}}{{\varvec{R}}}_{{\varvec{m}}{\varvec{a}}{\varvec{x}}}-\boldsymbol{ }{\varvec{G}}{{\varvec{R}}}_{{\varvec{m}}{\varvec{i}}{\varvec{n}}}}$$

Here, GR_log_ is the log reading, and GR_min_ and GR_max_ are the average values for clean sand and pure shale, respectively.

The shale volume (Vsh) was computed from the Gamma Ray (GR) log using the linear method. To ensure accuracy, the log-derived Vsh values were calibrated against XRD-measured clay contents from conventional core samples in the Simian-1 and Simian-2 wells. Depth matching was performed between the log data and XRD sample depths, and a cross-plot was constructed to evaluate the correlation between the two datasets.

#### Clay mineral identification

##### Clay mineral identification via spectral gamma ray cross-plots

The accurate identification of clay minerals is a fundamental step in petrophysical evaluation due to their significant influence on reservoir properties, including porosity, permeability, and fluid saturation. Different clay types possess distinct cation exchange capacities and morphologies, which directly affect these properties. In this study, clay mineralogy was determined through the analysis of spectral gamma ray log data.

The methodology is based on the characteristic radioactive responses of key clay minerals, primarily driven by their potassium (K) and thorium (Th) content^24^. Specific clay minerals, such as illite and mica, are potassium-rich, while others, like kaolinite and chlorite, are typically associated with thorium. Smectite often exhibits low levels of both elements.

A series of cross-plots were generated within the TechLog software to differentiate these mineralogical signatures. The primary cross-plot employed for clay minerals identifications was the Thorium (Th) vs. Potassium (K). The interpretation of these cross-plots was rigorously calibrated against X-ray diffraction (XRD) analysis performed on core samples from the studied interval.

##### Quantification of clay distribution using the Thomas-Stieber model

Beyond mineral identification, the spatial distribution of clay within the pore system is a critical control on reservoir quality. Laminated, structural, and dispersed shale each have a profoundly different effect on porosity–permeability relationships and effective hydrocarbon pore volume.

To quantify the type of clay distribution, the Thomas-Stieber model was applied^25^. This model establishes a deterministic relationship between gamma-ray log response (used as a clay volume indicator, Vsh), total porosity (ϕt), and the resulting clay distribution mode. The cross-plot of Vsh vs. ϕt allows to determine which clay type is dominant within the Simian reservoir from the following types:

- Laminated shale: Discrete layers of shale within the reservoir sand.
- Dispersed shale: Clay particles occupying the pore space of a sand framework.
- Structural shale: Clay particles replacing sand grains in the rock matrix.

This distribution has direct implications for calculating correct water saturation and estimating original hydrocarbon in place, as dispersed clay can significantly complicate resistivity-based saturation models^22,26^.

#### Porosity calculation

Porosity was calculated from the density log and corrected for shale content and gas effect to obtain the effective porosity (**ϕeff)** and the reported porosity in this study therefore represents the interconnected pore volume:

Total porosity (${{\varvec{\phi}}}_{{\varvec{t}}}$) was calculated from the Density Log using the equation:

$${{\varvec{\phi}}}_{{\varvec{t}}}=\frac{\left({{\varvec{\rho}}}_{{\varvec{m}}{\varvec{a}}}-\boldsymbol{ }{{\varvec{\rho}}}_{{\varvec{b}}}\right)}{\left({{\varvec{\rho}}}_{{\varvec{m}}{\varvec{a}}}-\boldsymbol{ }{{\varvec{\rho}}}_{{\varvec{f}}}\right)}$$

where ρma is the matrix density (2.65 g/cm^3^ for sandstone), ρb is the bulk density from the log, and ρf is the pore fluid density (adjusted for gas effect). The resulting effective porosity was then corrected for shale content:

$${{\varvec{\phi}}}_{{\varvec{e}}{\varvec{f}}{\varvec{f}}}=\boldsymbol{ }{{\varvec{\phi}}}_{{\varvec{t}}}-\boldsymbol{ }\left({{\varvec{V}}}_{{\varvec{s}}{\varvec{h}}}\times {{\varvec{\phi}}}_{{\varvec{s}}{\varvec{h}}}\right)$$

$\text{where }{{\varvec{\phi}}}_{{\varvec{e}}{\varvec{f}}{\varvec{f}}}$ is the effective porosity and ${{\varvec{\phi}}}_{{\varvec{s}}{\varvec{h}}}$ is the porosity of the adjacent shale zone.

#### Fluid saturation

The Archie equation^27^ was used to calculate water saturation (Sw) in the clean, clay-free intervals:

$${{\varvec{S}}}_{{\varvec{w}}}^{{\varvec{n}}}=\frac{\left({\varvec{a}}\times {{\varvec{R}}}_{{\varvec{w}}}\right)}{\left({{\varvec{\phi}}}^{{\varvec{m}}}\times {{\varvec{R}}}_{{\varvec{t}}}\right)}$$

where:

- Rt = True formation resistivity from deep resistivity log (Ω.m)
- Rw = Formation water resistivity (Ω.m)
- a = Tortuosity factor (usually 1)
- m = Cementation exponent (typically 2)
- n = Saturation exponent (typically 2)

For shaly sand intervals, the Simandoux equation^28^ was employed to account for the conductivity of shale:

$$\frac{1}{{{\varvec{R}}}_{{\varvec{t}}}}=\frac{{{\varvec{V}}}_{{\varvec{s}}{\varvec{h}}}\times {{\varvec{S}}}_{{\varvec{w}}}}{{{\varvec{R}}}_{{\varvec{s}}{\varvec{h}}}}+\frac{{{\varvec{\phi}}}_{{\varvec{e}}{\varvec{f}}{\varvec{f}}}^{{\varvec{m}}}\times {{\varvec{S}}}_{{\varvec{w}}}^{{\varvec{n}}}}{{\varvec{a}}\boldsymbol{ }\times {{\varvec{R}}}_{{\varvec{w}}}\left(1\boldsymbol{ }-\boldsymbol{ }{{\varvec{V}}}_{{\varvec{s}}{\varvec{h}}}\right)}$$

Hydrocarbon saturation was then calculated as Sh = 1—Sw.

### Determination of the gas–water contact

Accurate delineation of the free water level (FWL) and the subsequent gas–water contact (GWC) is a critical prerequisite for petrophysical interpretation, as it establishes the fundamental zonal discrimination between productive hydrocarbon-bearing intervals (“pay zones”) and water-saturated non-pay zones.

**Fig. 3:**
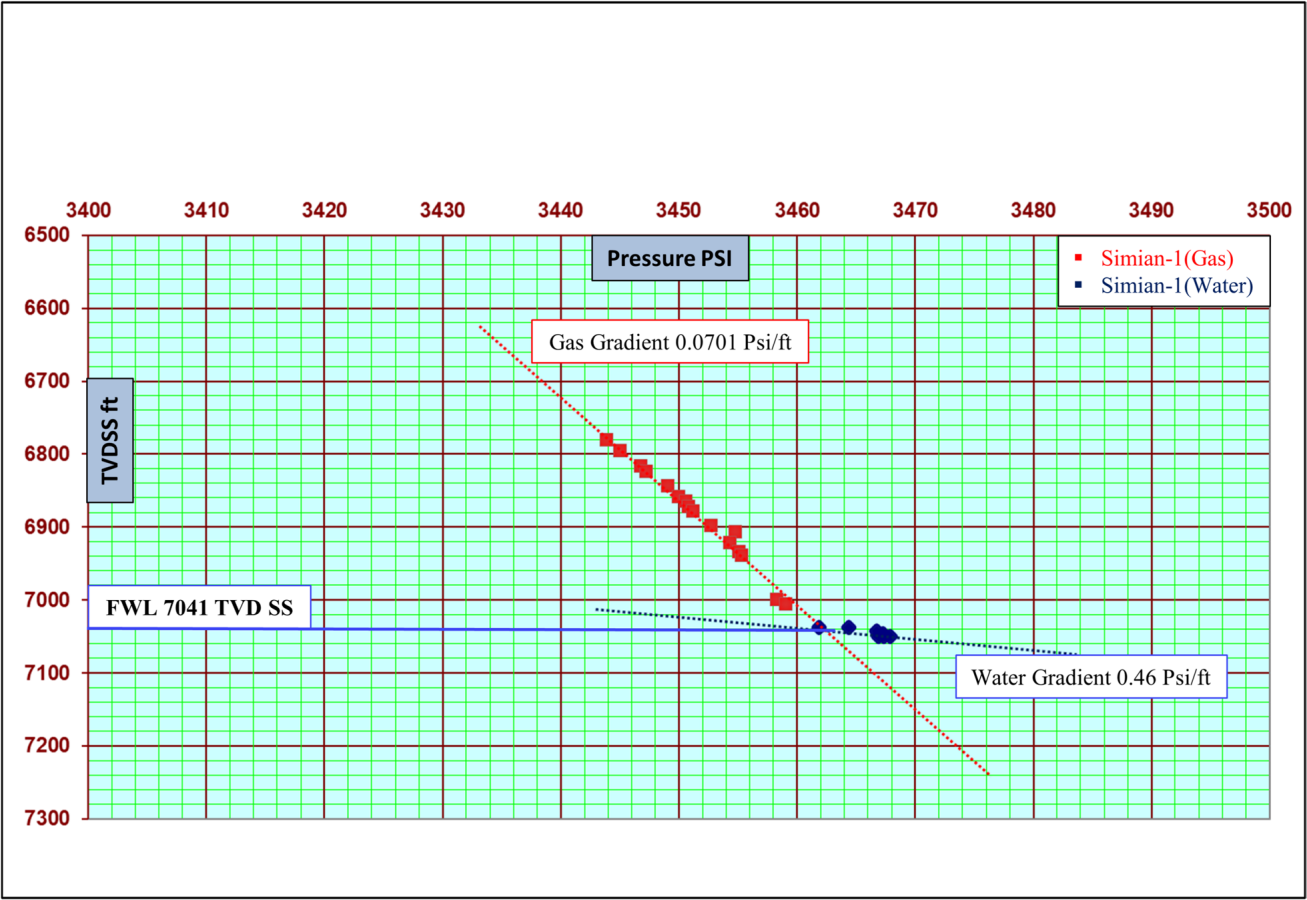
Determination of the gas–water contact (GWC) and free-water level (FWL) from modular formation dynamics tester (MDT) pressure measurements in the Simian-1 well. The plot shows measured pressure (psi) versus true vertical depth subsea (TVDSS, ft), with distinct gas (0.065 psi/ft) and water (0.46 psi/ft) gradients intersecting at the FWL, identified at approximately 7041 ft (2146 m) TVDSS. The discrete data points are characteristic of MDT pressure measurements, which are strategically acquired at selected depths to define the fluid gradients.

In this study, the definitive identification of the GWC was achieved through the analysis of formation pressure data acquired by a Modular Formation Dynamics Tester (MDT) tool. Pressure measurements were taken at discrete depths throughout the interval of reservoir in simian-1 well only (some pressure point in gas and others in water zone). These data were used to construct hydrostatic pressure gradients for both the gas and water columns (Fig. 3).

The GWC is explicitly defined as the depth at which the extrapolated gas and water pressure gradients intersect, indicating a state of capillary equilibrium. This pressure-derived contact provides a direct and quantitative measurement of the free water level.

### Formation water resistivity (Rw) determination

A critical and foundational step prior to petrophysical interpretation is the accurate determination of formation water resistivity (Rw). An erroneous Rw value introduces significant systematic error into all subsequent calculations of water saturation (Sw) via Archie’s equation and related models.

**Fig. 4:**
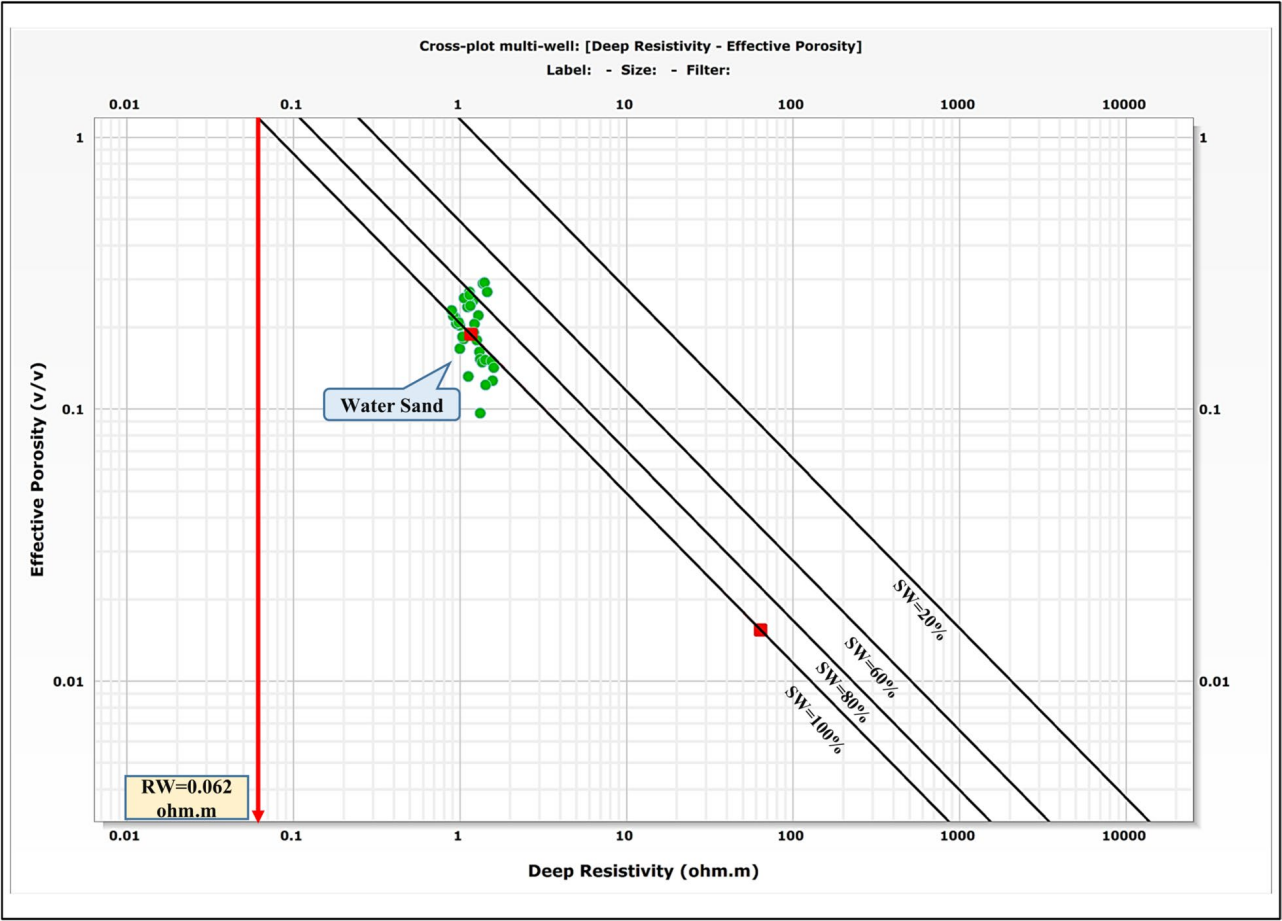
Pickett plot for the determination of formation water resistivity (Rw) in Simian-1 well. The log–log crossplot of effective porosity (φeff) versus true formation resistivity (Rt) in the water-bearing zone yields a trendline whose intercept at 100% porosity gives an Rw value of 0.062 Ω·m, consistent with direct MDT sample measurements RW.

In this study, Rw was constrained using two independent and complementary methods to ensure robustness and accuracy:

- Direct measurement from MDT fluid samples: Formation water was sampled directly downhole using the modular formation dynamics tester (MDT) tool. The resistivity of these recovered water samples was measured under laboratory conditions at a specific temperature. This measured value was then corrected to represent the in-situ Rw at the reservoir’s true formation temperature using the standard Arps equation.
- Graphical determination via pickett plot analysis: The Pickett plot, a log–log crossplot of porosity (ϕ) versus true formation resistivity (Rt), was employed as an apparent method. In water-bearing zones, data points align along a trendline with a slope equal to the cementation exponent (m). The extrapolation of this trendline to 100% porosity (ϕ = 1.0) provides the value for Rw (Fig. 4).

### Core data analysis

Conventional core analysis data from the corresponding depth intervals were used to calibrate and validate the log-derived measurements.

#### Thin section interpretation

A multi-scale analytical approach was employed to characterize the mineralogy and pore system of the reservoir rocks, with a particular focus on clay mineralogy due to its fundamental control on petrophysical properties. The accurate identification of clay phases—including illite, illite/smectite mixed-layer (I/S), chlorite, and kaolinite—is critical, as their type, morphology, and distribution exert a primary influence on porosity, permeability, and capillary properties, thereby governing hydrocarbon storage and flow dynamics. For example, pore-lining fibrous illite is known to occlude pore throats and drastically reduce permeability, while authigenic chlorite coatings can inhibit quartz overgrowths, thereby preserving primary porosity^29–33^.

Scanning electron microscopy (SEM) was utilized to identify these clay minerals and analyze their micro-scale distribution, morphology, and paragenetic relationships. This technique provides the high-resolution imaging necessary to diagnose distinctive clay textures, such as the honeycomb morphology of kaolinite or the fibrous habit of illite.

Furthermore, the analysis was integrated with traditional petrographic methods to provide a comprehensive assessment of reservoir quality. Detailed thin section petrography was conducted to determine mineral composition, texture, and key diagenetic features such as cementation and dissolution, all of which directly impact porosity and permeability^34^. Complementarily, grain size distribution analysis was performed to quantify sorting and packing characteristics, which are fundamental parameters controlling pore connectivity and fluid flow behavior.

A total of 50 thin sections from representative core plugs were analyzed under a polarizing microscope. This analysis provided detailed descriptions of mineral composition (e.g., quartz, feldspar, clay minerals), texture, grain size and sorting, diagenetic features (cementation, dissolution), and pore types, offering a microscopic ground-truth for the log-based lithology.

#### Porosity calibration

Core-derived porosity, measured using a helium porosimeter, was directly compared to the log-derived effective porosity. A linear regression function was established to calibrate the log porosity, thereby improving the accuracy of porosity estimates in uncored intervals and wells.

### Rock typing

Hydraulic Flow Units (HFUs) were identified to classify the reservoir into rock types with similar pore-throat characteristics and fluid flow behavior.

#### Reservoir quality index (RQI) and flow zone indicator (FZI)

Based on the modified Kozeny-Carman model^35,36^, rock typing was performed on the core data using the following steps:

- **Reservoir quality index (RQI)** was calculated (in microns) for each core sample:
  $$\mathbf{R}\mathbf{Q}\mathbf{I}=0.0314\sqrt{{\varvec{K}}/{\varvec{\phi}}}$$

where *K* is air permeability (mD) and **ϕ** is core porosity (fraction).

- Normalized porosity index (ϕz) was calculated as:
  $${{\varvec{\phi}}}_{{\varvec{z}}}=\frac{{{\varvec{\phi}}}_{\boldsymbol{ }}}{1\boldsymbol{ }-\boldsymbol{ }{\varvec{\phi}}}$$
- The **flow zone indicator (FZI)** is derived from:
  $${\varvec{F}}{\varvec{Z}}{\varvec{I}}\boldsymbol{ }=\frac{{\varvec{R}}{\varvec{Q}}{\varvec{I}}}{{{\varvec{\phi}}}_{{\varvec{z}}}}$$

On a log–log plot of RQI versus **ϕz**, samples with similar FZI values cluster together, defining distinct HFUs.

#### Stratigraphic modified Lorenz (SML) plot

The analysis of hydraulic flow units (HFUs) within the reservoir interval was conducted using the Stratigraphic Modified Lorenz (SML) plot method. This technique provides a robust, data-driven approach to subdivide a reservoir into distinct hydraulic units based on the pore-throat characteristics of the rock, rather than relying solely on traditional lithostratigraphic boundaries or porosity–permeability transforms^37^.

As a complementary technique, the SML plot was used to identify flow units based on the storage and flow capacity of the reservoir. This involves:

- Calculating **flow capacity** (K * h) and **storage capacity** (ϕ* h) for each depth interval.
- Computing the cumulative percent of both flow and storage capacity from the top of the reservoir downward.
- Plotting cumulative flow capacity vs. cumulative storage capacity.

The resulting plot is used to segment the reservoir into intervals with similar flow characteristics (slope of the curve), where a change in slope indicates a boundary between hydraulic units.

The core-based FZI analysis provides a definitive rock typing scheme at the cored wells. To extend this classification to uncored wells across the field, the established relationship between HFUs and conventional well logs can be leveraged. Predictive models, such as supervised neural network classifiers, can be trained using the core-calibrated HFUs as the target and the suite of environmental-corrected logs (Gamma Ray, Resistivity, Neutron, Density) as inputs. While this predictive application is a critical next step for full-field reservoir modeling, it is considered a subsequent phase of work that builds upon the core-based characterization framework presented here.

## Results

### Fluid properties and contacts

Formation pressure data acquired through Modular Formation Dynamics Tester (MDT) measurements reveal a hydrostatically connected sand system throughout the Simian-1 well. Analysis of pressure versus depth plots demonstrates a consistent gas gradient of 0.13 psi/ft, extending down to 2146 m TVDSS, below which a definitive gradient transition to 0.46 psi/ft establishes the Free Water Level (FWL) at 2146 m TVDSS (2161 m MD) (Fig. 3).

To corroborate this pressure-based interpretation and to characterize the transition zone, the identified GWC was integrated with and calibrated against wireline log signatures. A sharp increase in resistivity, coupled with a prominent “gas crossover” effect between neutron and density porosity curves, provides strong petrophysical evidence for gas-bearing zones above the contact. The integration of these independent datasets—direct pressure measurements and log responses—ensures a robust and accurate definition of the fluid contacts for subsequent volumetric and saturation analysis.

Furthermore, integration of pressure and structural data across all studied wells indicates lateral variation in the GWC and gas-down-to levels. The Simian-1, Simian-2, and Simian-3 wells share a hydraulically connected gas system with consistent pressure gradients, confirming they belong to the same gas-bearing compartment within the Simian Main Channel. In contrast, the Simian-Dr well shows a different pressure regime and deeper water contact (around 2185 m TVDSS), implying partial hydraulic isolation related to the West Channel System (Fig. 3).

Formation water resistivity (Rw) determination using Pickett plot methodology yields a value of 0.062 Ohm.m at formation temperature (Fig. 4). This value shows remarkable consistency with Rw measurements of 0.065 Ohm.m obtained from direct analysis of MDT water samples, providing robust confirmation of formation water characteristics and validating the log-derived saturation models. The exceptional concordance between the direct laboratory measurement (0.065 Ω·m) and the value derived from the petrophysical signature of the reservoir (0.062 Ω·m) provides a high degree of confidence in the selected Rw. This strong correlation validates the petrophysical model parameters and confirms the representativeness of the acquired fluid sample.

### Lithology, mineralogy, and shale distribution

**Fig. 5:**
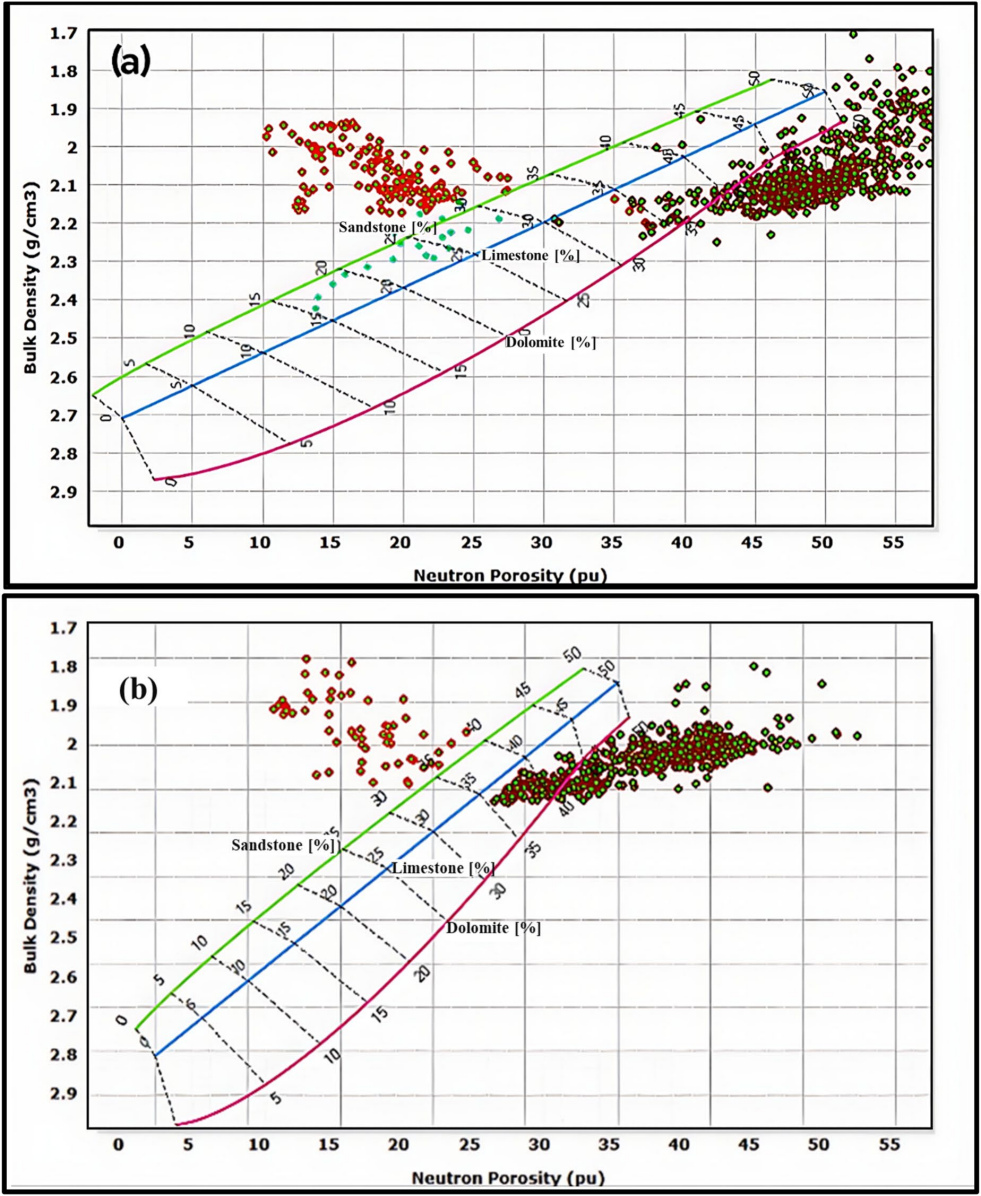
Neutron-density cross-plot for lithology identification in Simian-1 and Simian-2 wells. It’s clear that the main lithology is sandstone interbeded with shale characterizing El Wastani Formation.

**Fig. 6:**
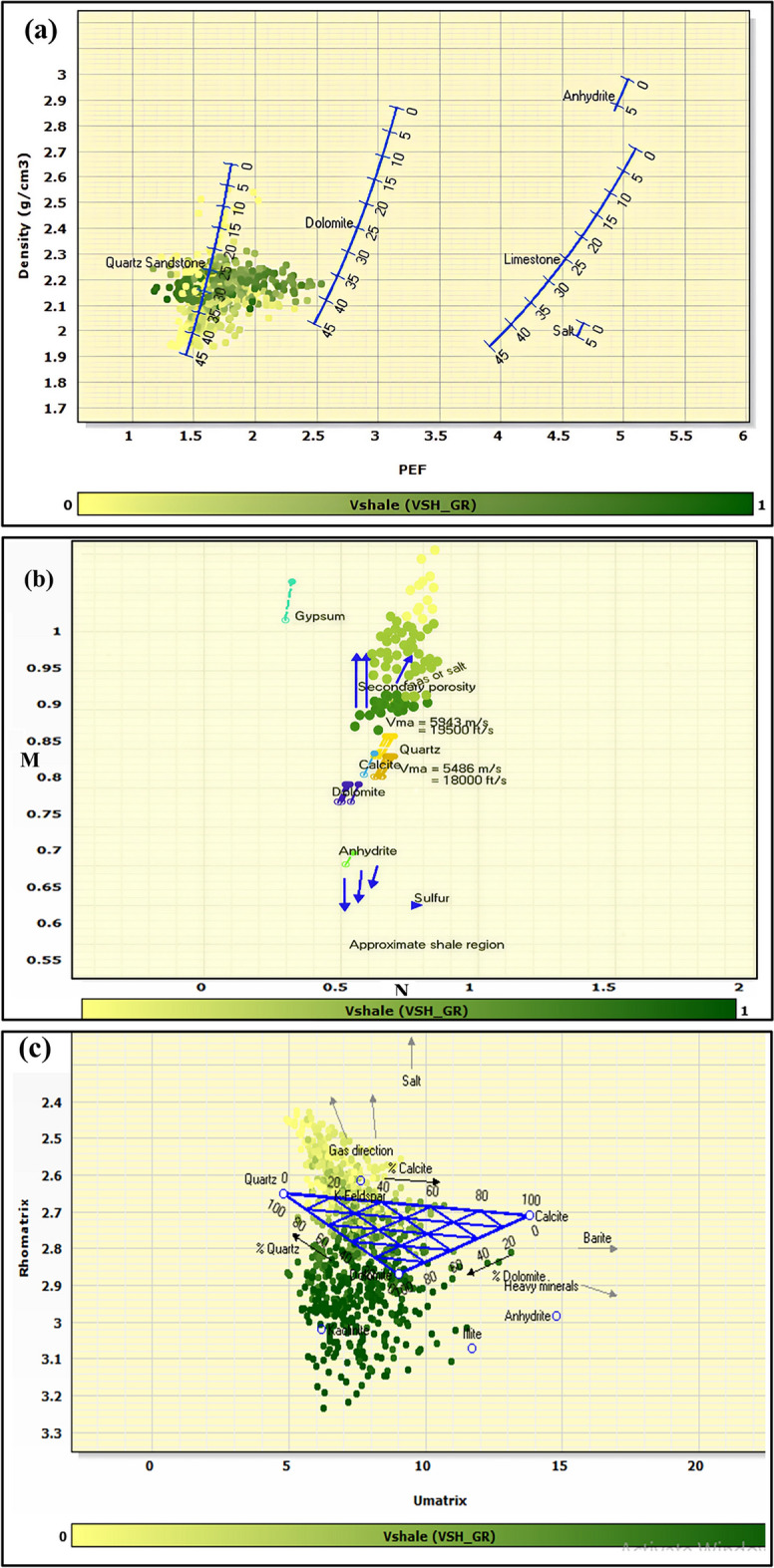
Supplementary lithology identification cross-plots for Simian-1 and Simian-2 wells. (**a**) Photoelectric Factor (PEF) versus bulk density (ρb). (**b**) M–N plot for complex mineralogy discrimination. (**c**) Umatrix versus Rhomatrix plot. All plots confirm the dominant sand-shale lithology.

Integrated petrophysical analysis of the El Wastani Formation in the Simian field reveals a complex clastic succession dominated by interbedded sand and shale formations. Comprehensive lithological identification employing multiple cross-plot techniques—including neutron-density (Fig. 5), PEF-density (Fig. 6a), M–N (Fig. 6b), and Umatrix-Rhomatrix (Fig. 6c) analyses—consistently confirms this lithological framework.

**Fig. 7:**
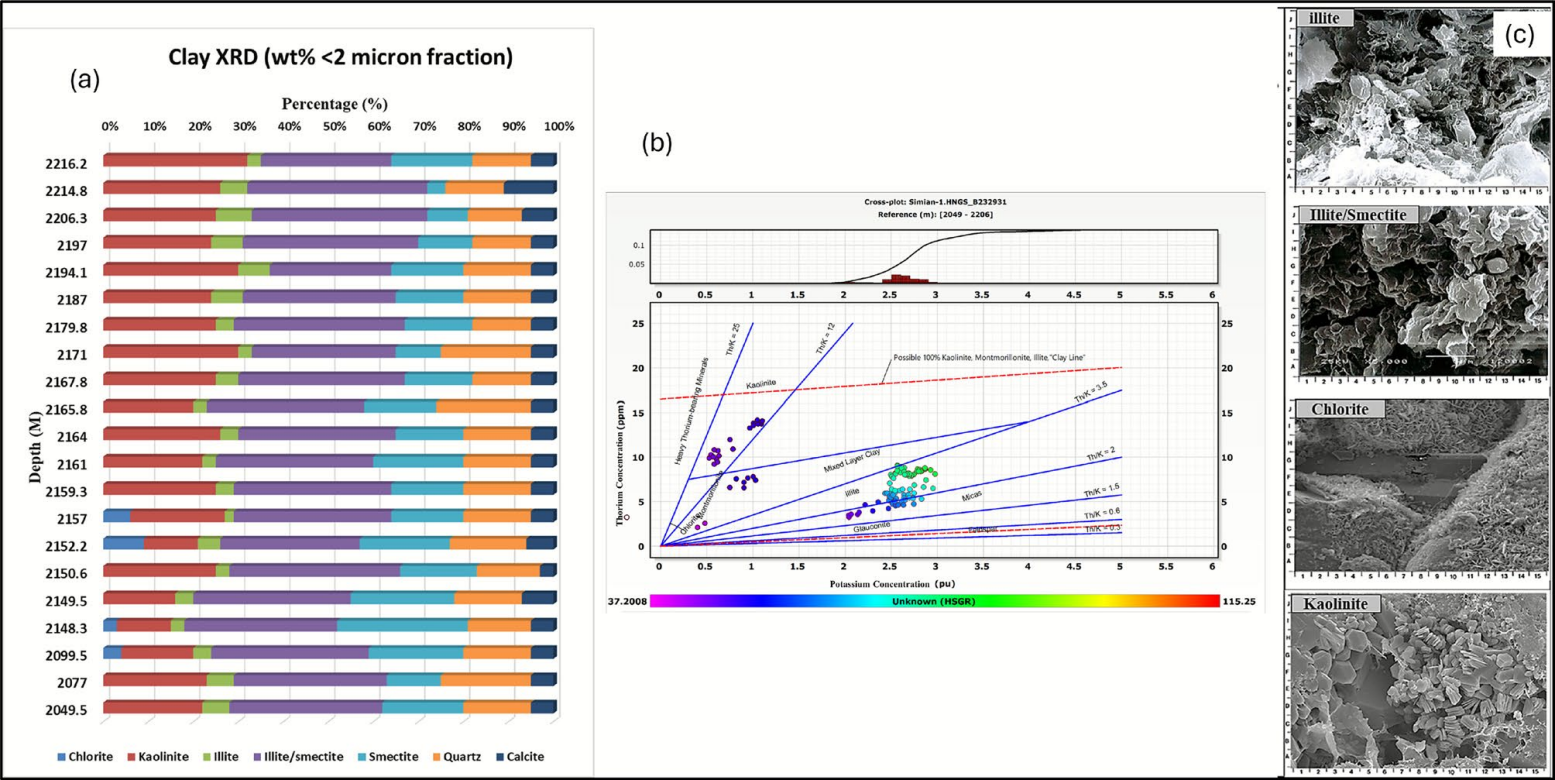
Clay mineralogy characterization for Simian-1 well. (**a**) Representative X-ray Diffraction (XRD) analysis results showing the relative percentages of clay minerals. (**b**) Thorium versus Potassium (Th-K) cross-plot from spectral gamma ray logs, used for clay typing. (**c**) Scanning Electron Microscopy (SEM) image illustrating the micro-scale distribution and morphology of clay minerals (e.g., illite/smectite) within the pore system.

**Fig. 8:**
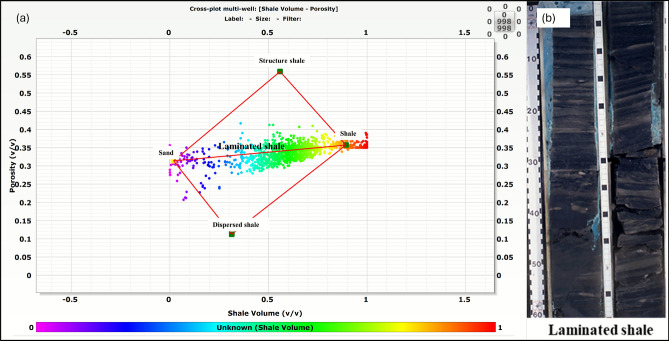
Analysis of shale distribution using the Thomas-Stieber model for Simian-1 and Simian-2 wells. (**a**) Cross-plot of total porosity (ϕt) versus shale volume (Vsh), defining that the dominant is laminated shale distribution mode. (**b**) Core photograph from a representative interval, visually confirming the presence of thin, continuous shale laminations within the reservoir sand.

Detailed clay mineralogy characterization, derived from potassium-thorium (Th-K) cross-plots (Fig. 7b) calibrated against core-based XRD and SEM analysis (Fig. 7a,c, repectively), identifies a complex mixed-layer assemblage dominated by smectite/illite with subordinate mica components within the Simian channel deposits. Clay mineralogy characterization through XRD and SEM analyses reveals a complex assemblage dominated by illite/smectite mixed-layer clay (55–60%), illite (20–25%), kaolinite (10–15%), and smectite (5–8%), with systematic variation between facies types (Fig. 8). This mineralogical distribution exerts fundamental control on reservoir quality through its influence on pore geometry and surface conductivity.

The Thomas-Stieber model analysis (Fig. 8a), validated through core photography (Fig. 8b), establishes laminated shale as the predominant structural type in Simian reservoir, with approximately 65% of shale intervals exhibiting well-developed laminations that significantly impact reservoir anisotropy. No clear evidence of dispersed or structural shale was identified from either log or core data.

Both shale volume (Vsh) and porosity results were calibrated using core data to ensure reliability. The Vsh values derived from the Gamma Ray log were calibrated against XRD-measured clay content, while the log-derived porosity was calibrated against helium porosity measurements from core analysis. Both calibrations showed a good overall match, confirming the consistency between log-derived and core-derived parameters.

**Fig. 9:**
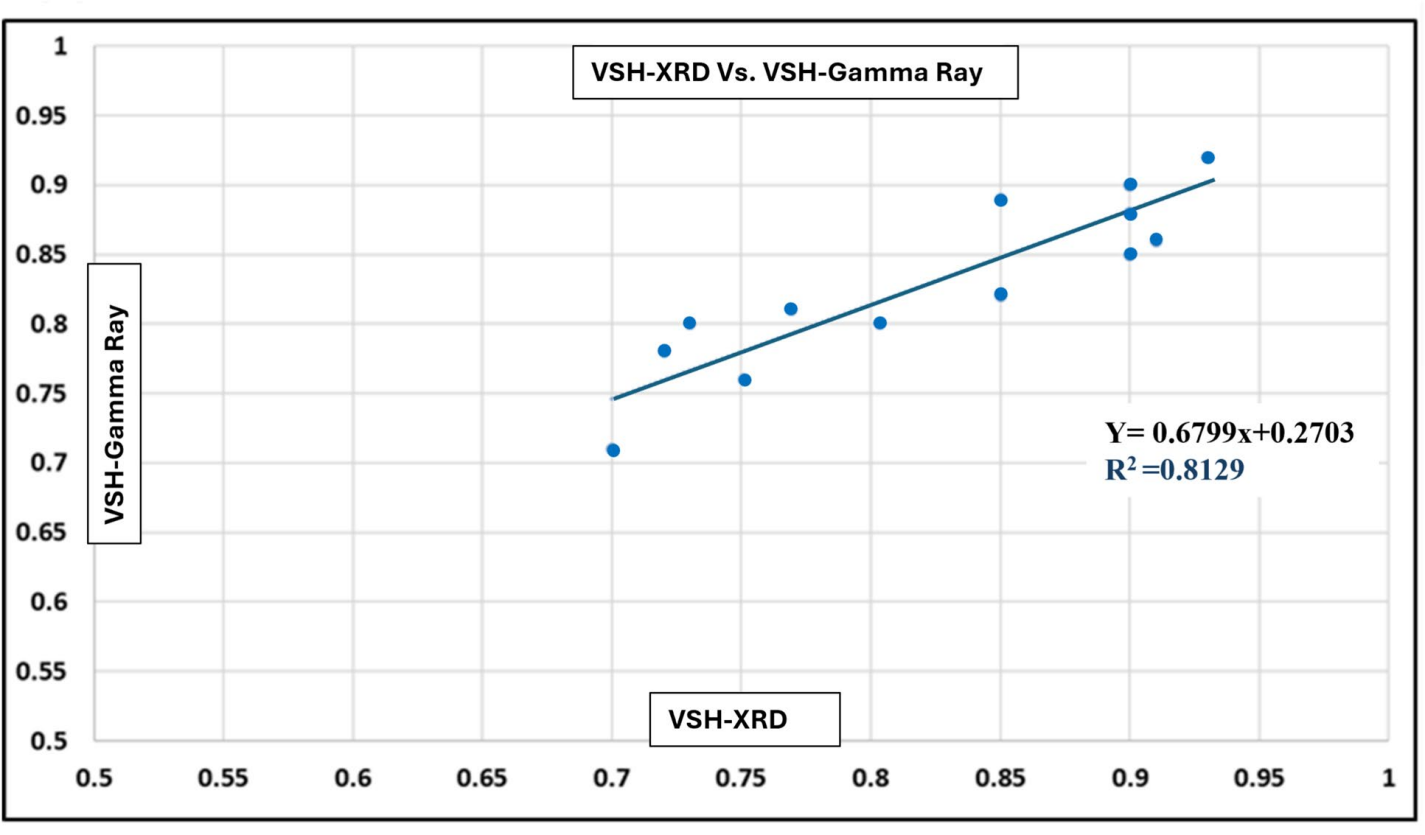
Cross-plot between Gamma Ray–derived shale volume (Vsh) and XRD-measured clay content from Simian-1 cores. The calibration shows a strong correlation (R^2^ = 0.81) and good overall agreement throughout the interval.

The calibration between GR-derived Vsh and XRD-measured clay content showed a good overall match across the studied interval, with a correlation coefficient (R^2^) of 0.81, indicating strong consistency (Fig. 9). Two data points were observed slightly off the main trend, likely due to the difference in vertical resolution between the high-resolution XRD data and the lower-resolution log measurements. Overall, the calibration confirms the reliability of the GR-based shale-volume estimation for the Simian reservoir.

Quantitative petrophysical evaluation of reservoir intervals indicates a shale volume (Vsh) range of 17–31%, with effective porosity (φeff) varying between 20–24% and water saturation (Sw) ranging from 27–41%. These parameters demonstrate excellent reservoir quality throughout the evaluated sections, with average values summarized in Table 2.

**Table 2:** The average values of the estimated petrophysical parameters of the studied reservoir.

Well name	Pay zone	Top (m)	Bottom (m)	Gross Thickness (m)	Net Thickness (m)	Net to Gross	Shale volume v/v	Effective Porosity v/v	Effective water saturation v/v
Simian-1	Gas Sand	2088	2169.22	81.22	52.65	0.65	0.27	0.21	0.38
Simian-2	Gas Sand	2103.6	2197.3	93.70	54.4	0.58	0.35	0.25	0.39
Simian-3	Gas Sand	2064.4	2223.55	159.11	80.54	0.50	0.32	0.21	0.47
Simian-Dr	Gas Sand	2197.5	2279.40	81.90	24.634	0.30	0.34	0.24	0.50

### Geological framework and depositional environments

**Fig. 10:**
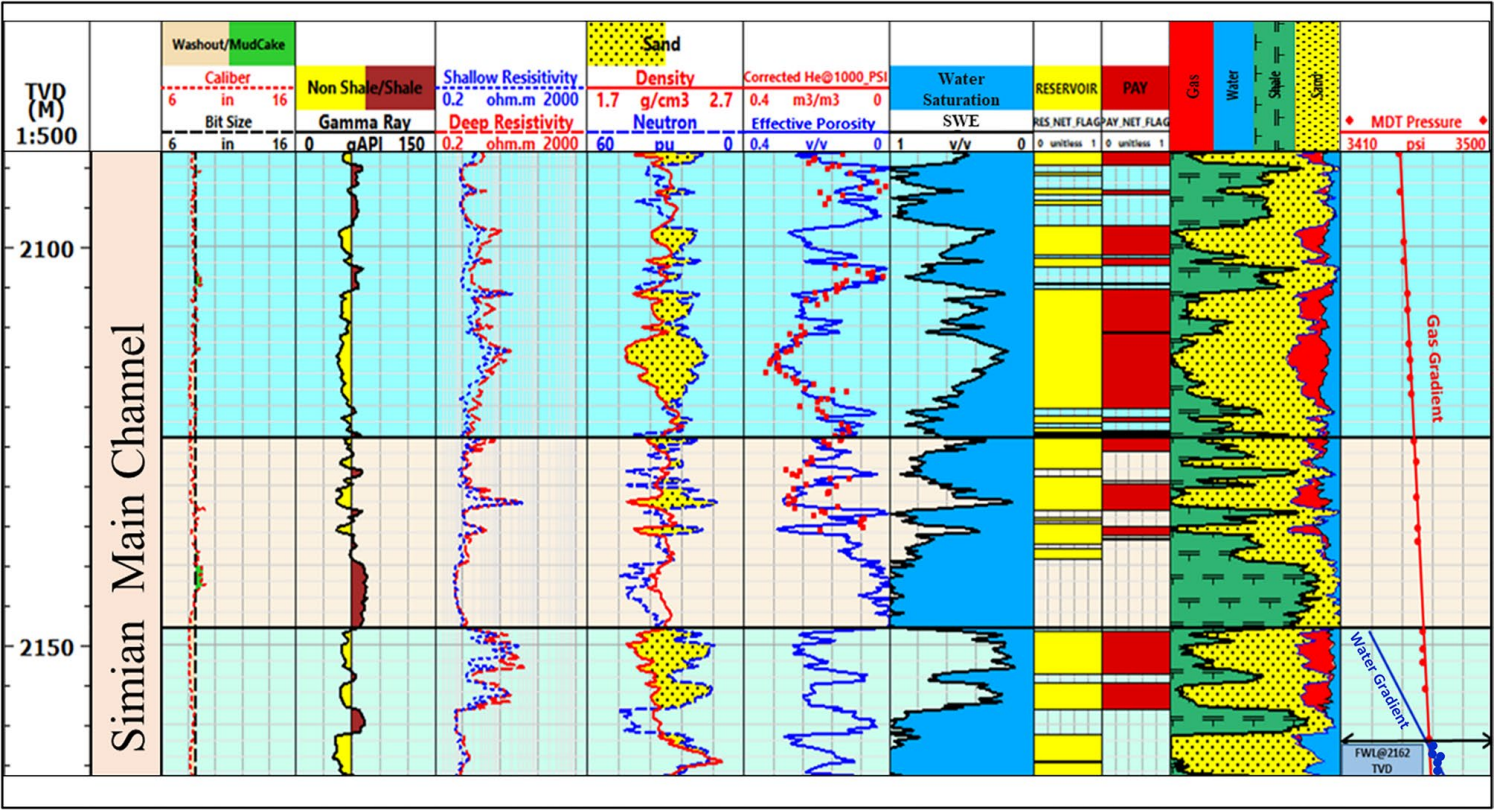
Composite litho-saturation plot for the Simian-1 well across the evaluated reservoir interval (2088.5–2198 m). Tracks typically include Gamma Ray (GR), deep resistivity (Rt), neutron total porosity (NPHI), bulk density (RHOB), calculated volumes of shale (Vsh), total porosity (PHIT), and water saturation (Sw), clearly showing the gas-bearing “pay zones” above the defined Free Water Level (FWL).

**Fig. 11:**
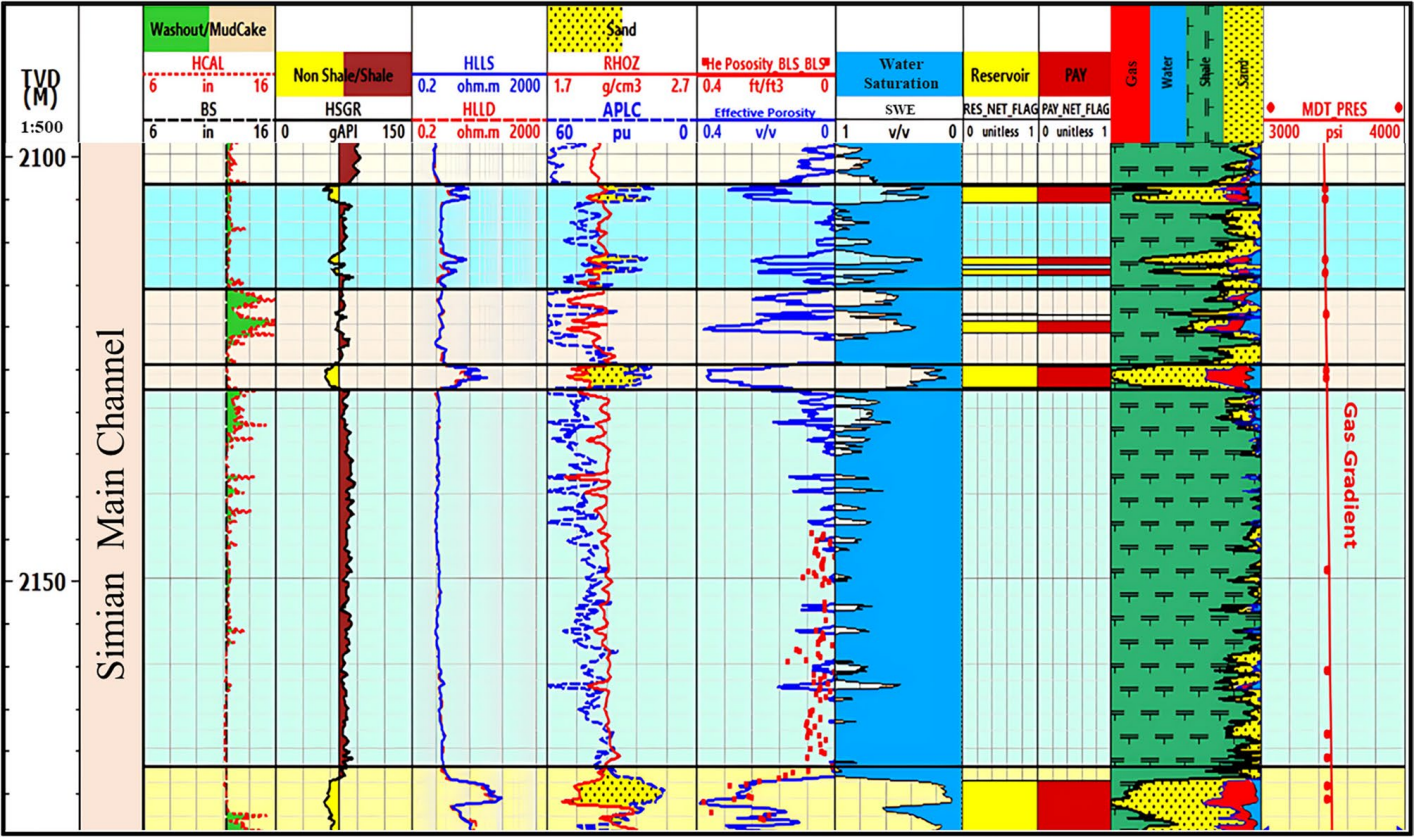
Litho-saturation plot for the Simian-2 well, illustrating reservoir properties and fluid distribution in a different part of the channel system.

**Fig. 12:**
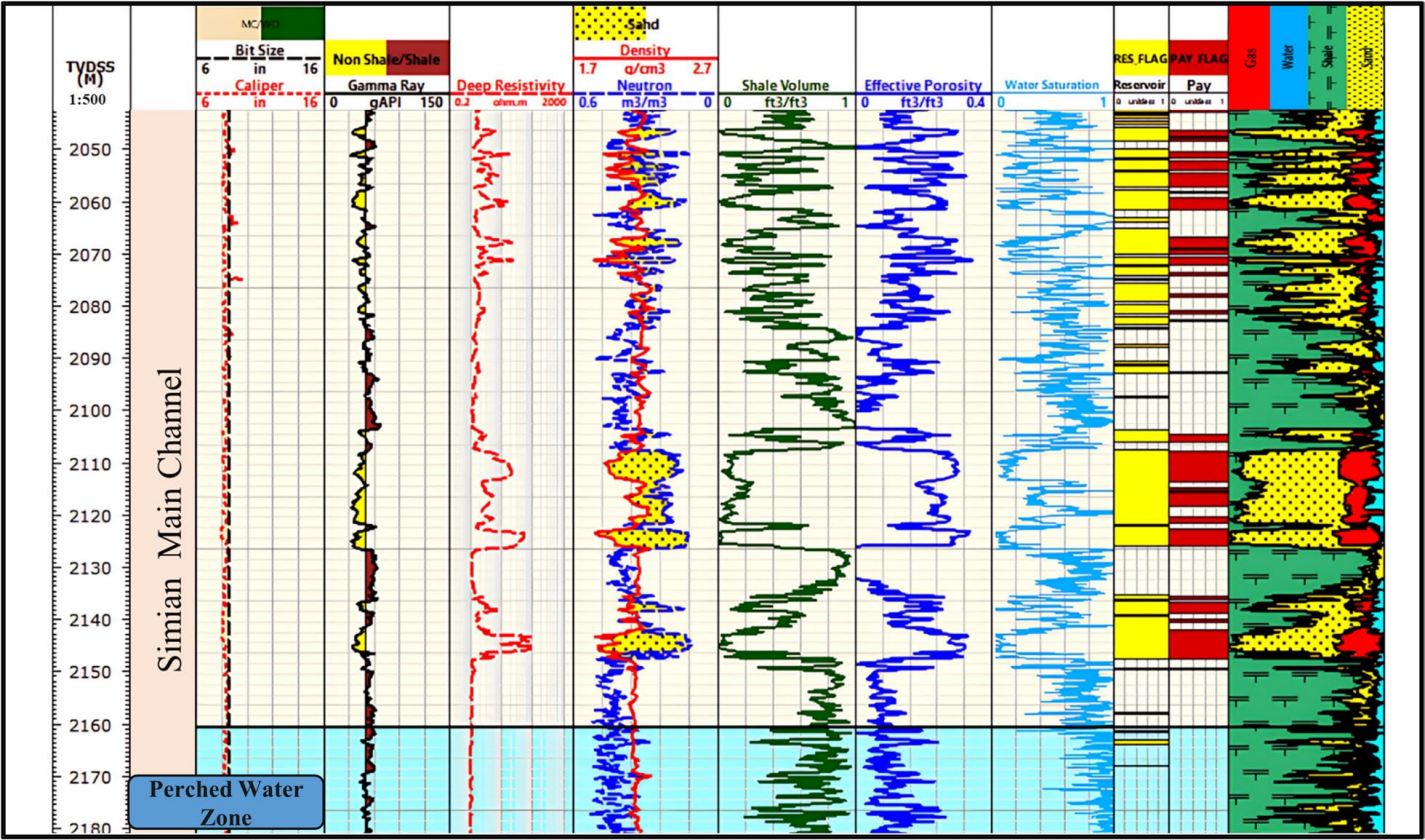
Litho-saturation plot for the Simian-3 well, providing further validation of the reservoir model and fluid contacts across the field.

**Fig. 13:**
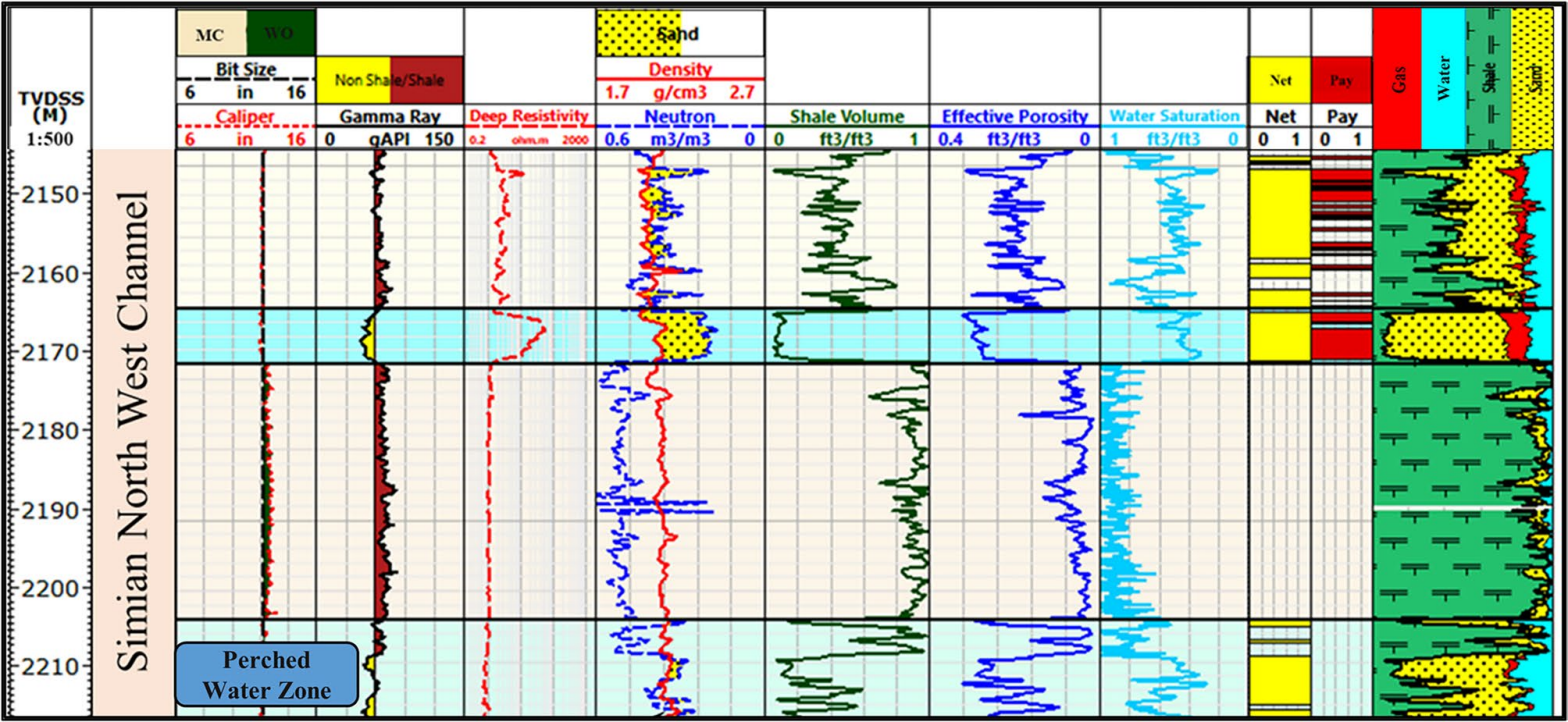
Litho-saturation plot for the Simian-Dr well, completing the multi-well analysis of the El Wastani Formation.

Analysis of the Simian-1 well, strategically located on the southern margin of the Simian channel system, elucidates the vertical distribution of hydrocarbons within a well-defined stratigraphic framework. The evaluated interval (2088.5–2198 m) encompasses a gross channel complex thickness of 78.4 m, with the channel top encountered at 2088 m. Litho-saturation cross-plots (Figs. 10–13) reveal the channel facies architecture composed of intercalated sandstone, shale, and siltstone units, with hydrocarbon saturation preferentially concentrated in the cleaner sandstone units positioned above the established FWL.

**Fig. 14:**
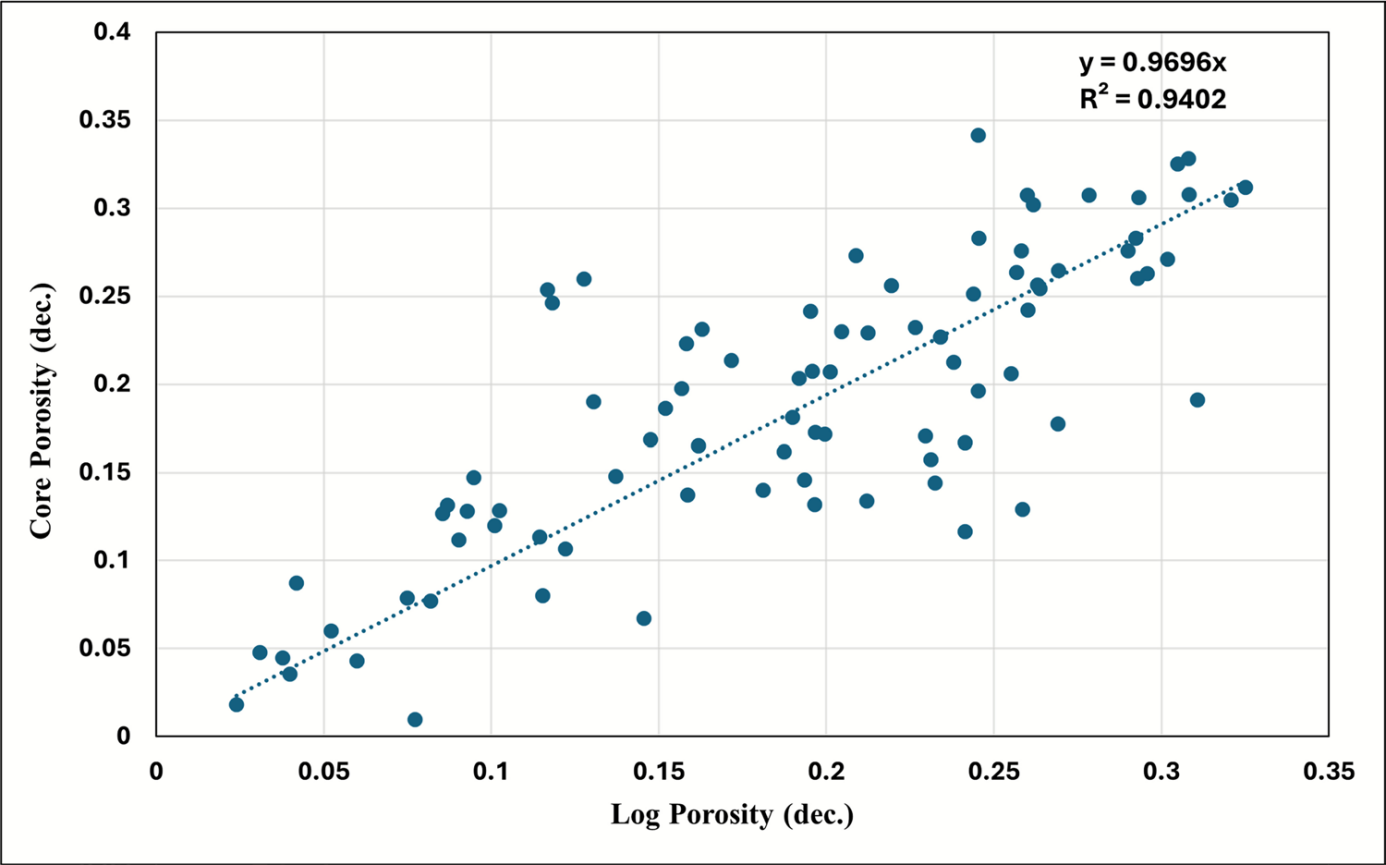
Core-to-log effective porosity calibration for Simian-1 and Simian-2 wells. Cross-plot of core-plug measured porosity versus log-derived effective porosity, demonstrating an excellent correlation (R^2^ = 0.94) and validating the petrophysical model.

Core-log integration established the fundamental basis for petrophysical validation through rigorous comparison of core-plug measurements with log-derived values. Regression analysis of paired core-log porosity data demonstrates exceptional correlation (R^2^ = 0.94, Fig. 14), providing robust quantitative validation of the log porosity algorithm and confirming the efficacy of data acquisition, environmental corrections, and processing methodologies.

**Fig. 15:**
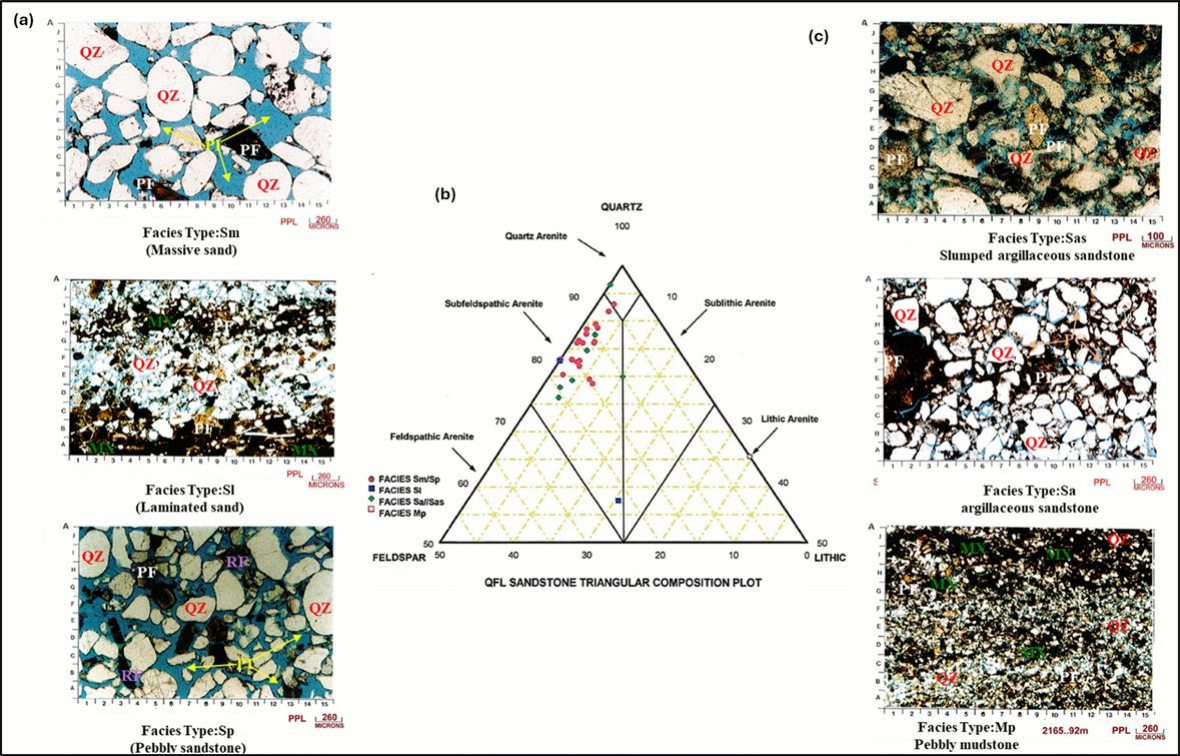
Petrographic analysis of reservoir rock composition for Simian-1 well. (**a**) Photomicrograph of a thin section showing detrital mineralogy. (**b**) Quartz-Feldspar-Lithic (QFL) triangular diagram classifying sandstone composition. (**c**) Another representative photomicrograph highlighting texture and diagenetic features.

Petrographic analysis reveals significant mineralogical heterogeneity within the detrital composition, particularly in the relative proportions of quartz (55–78%), feldspar (8–15%), and lithic fragments (12–22%) across the sedimentary succession (Fig. 15a,c) and the QFL sandstone triangular composition (Fig. 15b). Detailed sedimentological analysis based on 208 core samples identifies six principal sedimentary facies.

### Facies associations and interpretations

- **Facies Sm/Sp: massive to pebbly sandstone**: Characterized by light to medium grey, poorly indurated, calcareous sandstone with medium to coarse-grained texture and moderate to good sorting. Contains abundant coarse quartz grains and pebble to cobble-grade lithic fragments, with rare sideritic bands and scattered bioclasts. Interpreted as high-energy channel-fill deposits within a fluvial-dominated system.
- **Facies Mp: pebbly mudstone**: Comprises light to dark grey, subblocky to blocky mudstone with microcarbonaceous and micromicaceous components. Fossiliferous content includes common bivalve and foraminiferid fragments, with scattered pebble-grade quartz grains in a mud matrix. Interpreted as lower-energy abandoned channel or overbank deposits.
- **Facies Sa/Sas: argillaceous sandstone/slumped sandstone**: Features light greenish grey, highly argillaceous, slightly calcareous sandstone with micaceous components. Contains coarse-grained quartz, bivalve fragments, and mud clasts within a fine-grained matrix. Interpreted as unstable delta-front deposits affected by soft-sediment deformation and slumping processes.
- **Facies Sl: laminated sandstone**: Consists of light grey, very fine-grained, well-sorted sandstone with distinct laminations (1 mm to 2 cm thickness). Exhibits sharp basal contacts and gradational tops, with local micro-deformation features. Interpreted as lower-flow-regime deposits in distal fan or lobe settings.

### Hydraulic flow unit characterization and permeability modeling

The reservoir’s hydraulic architecture was rigorously delineated using Flow Zone Indicator (FZI) methodology applied to 208 core samples. Cluster analysis identified six distinct Hydraulic Flow Units (HFUs 1–6) representing unique pore systems with progressively increasing reservoir quality from HFU-1 (Mean RQI = 2.001) to HFU-6 (Mean RQI = 63.294) (Table 3).

**Table 3:** Hydraulic flow unit (HFU) permeability transform equations and statistical validation using reservoir quality index (RQI).

HFU	Mean RQI	Equation	R2
HFU 1	2.001	Perm = Phi3 * (2.001/(0.0314 * (1-Phi))) 2	0.70
HFU 2	4.159	Perm = Phi3 * (4.159/(0.0314 * (1-Phi))) 2	0.94
HFU 3	7.116	Perm = Phi3 * (7.116/(0.0314 * (1-Phi))) 2	0.98
HFU 4	11.204	Perm = Phi3 * (11.204/(0.0314 * (1-Phi))) 2	0.96
HFU 5	17.853	Perm = Phi3 * (17.853/(0.0314 * (1-Phi))) 2	0.91
HFU 6	63.294	Perm = Phi3 * (63.294/(0.0314 * (1-Phi))) 2	0.87

**Fig. 16:**
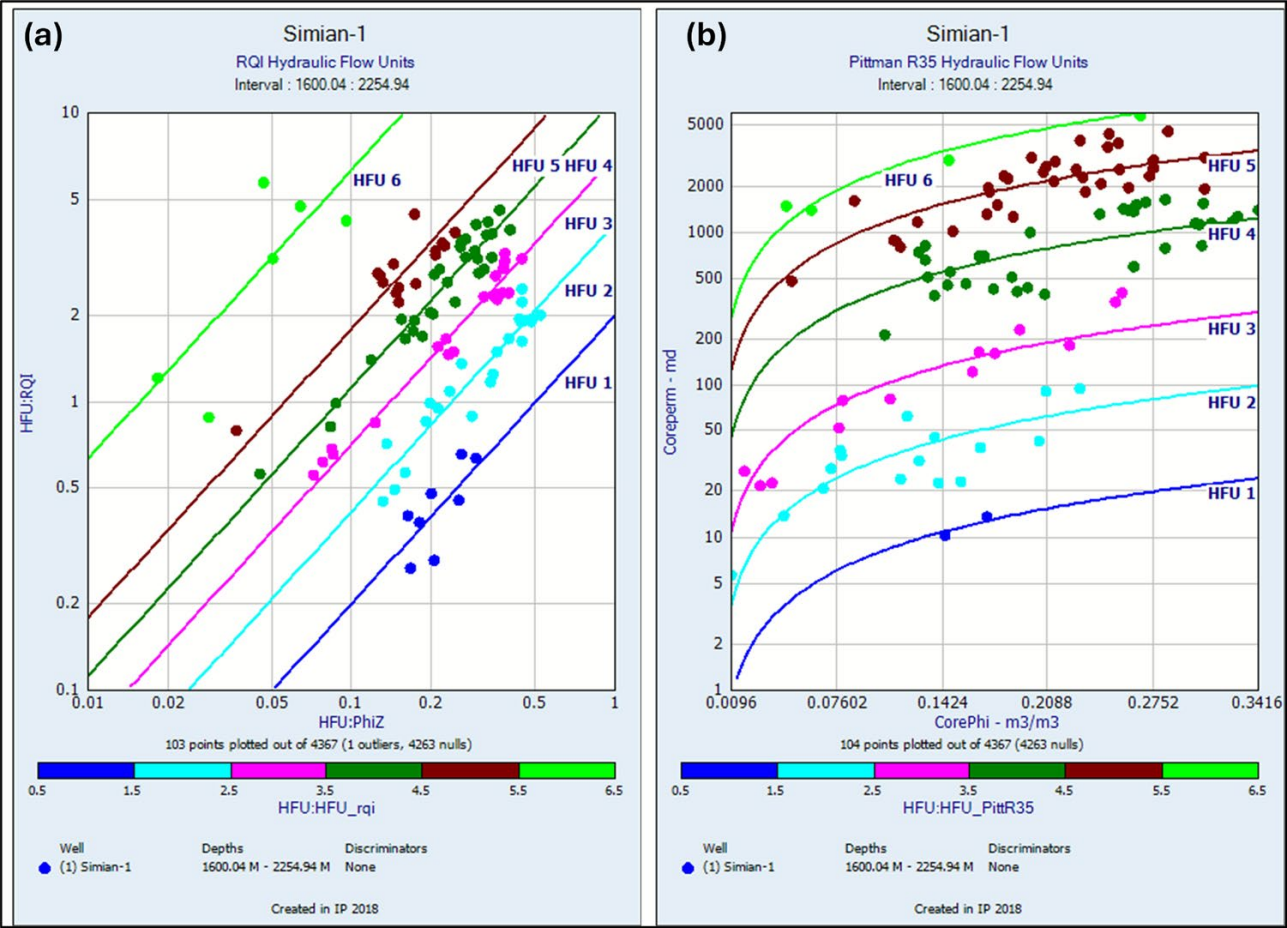
Hydraulic flow unit (HFU) classification for Simian-1 and Simian-2 wells. (**a**) Log–log plot of reservoir quality index (RQI) versus Normalized effective porosity Index (ϕz) identifying six distinct clusters (HFU-1 to HFU-6) based on the Flow Zone Indicator (FZI). (**b**) Equivalent rock typing plot using the Pittman R35 method for comparative analysis.

**Fig. 17:**
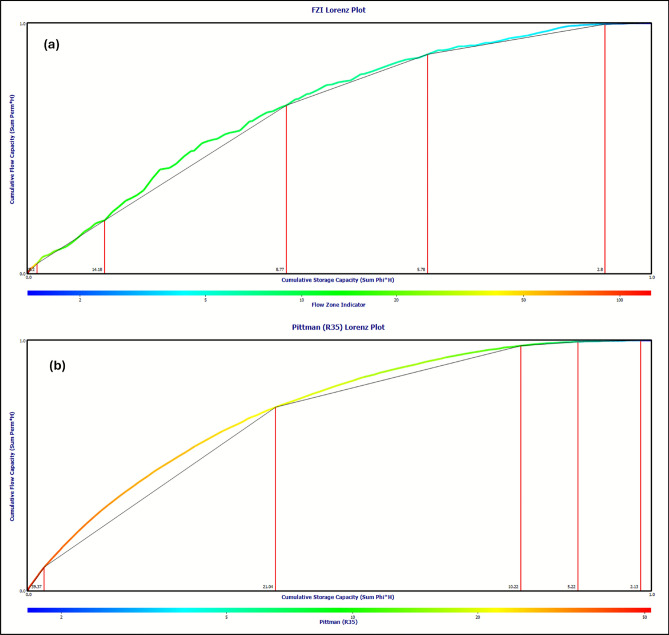
Stratified modified Lorenz (SML) plots for hydraulic zonation validation in Simian-1 and Simian-2 wells. (**a**) SML plot based on FZI-derived HFUs, showing the cumulative flow capacity versus cumulative storage capacity and segmenting the reservoir into six hydraulic units. (**b**) SML plot based on the Pittman R35 classification for comparison.

This classification is substantiated by multiple independent analyses: RQI versus normalized porosity plots demonstrate six unique trends (Fig. 16a), while Stratified Modified Lorenz (SML) analysis reveals six discrete segments in the flow-storage capacity profile (Fig. 17a), confirming the hydraulic zonation. The statistical robustness of the unit-specific porosity–permeability transforms is evidenced by consistently high coefficients of determination (R^2^ values ranging from 0.70 to 0.98, Table 3), with HFU-3 (R^2^ = 0.98) and HFU-4 (R^2^ = 0.96) showing exceptional predictive power.

Comparative validation using the Pittman R35 technique confirmed the six-HFU scheme (Table 4) but yielded transforms with greater variation in predictive accuracy (R^2^ = 0.54–0.93, Table 4) (Fig. 16b), while Stratified Modified Lorenz analysis based on the Pittman R35 technique reveals six discrete segments in the flow-storage capacity profile (Fig. 17b). The superior performance of RQI-derived transforms, particularly for the most productive intervals (HFU-3 to HFU-6), established them as the preferred methodology for permeability prediction and subsequent reservoir modeling.

**Table 4:** Hydraulic flow unit (HFU) permeability transform equations and statistical validation using Pittman R35 hydraulic flow units technique.

HFU	Mean RQI	Equation	R2
HFU 1	1.727	Perm = 10^((Log(1.727)—0.255 + 0.523*Log(Phi))/0.565)	0.80
HFU 2	3.788	Perm = 10^((Log(3.788)—0.255 + 0.523*Log(Phi))/0.565)	0.67
HFU 3	7.085	Perm = 10^((Log(7.085)—0.255 + 0.523*Log(Phi))/0.565)	0.90
HFU 4	15.767	Perm = 10^((Log(15.767)—0.255 + 0.523*Log(Phi))/0.565)	0.54
HFU 5	28.083	Perm = 10^((Log(28.083)—0.255 + 0.523*Log(Phi))/0.565)	0.65
HFU 6	43.937	Perm = 10^((Log(43.937)—0.255 + 0.523*Log(Phi))/0.565)	0.93

The HFU distribution shows strong correlation with sedimentary facies, with HFU-4 and HFU-5 predominantly associated with Facies Sm/Sp, while HFU-1 and HFU-2 correlate with Facies Mp and Sa/Sas. This relationship between depositional environment and pore architecture provides crucial insights for reservoir distribution prediction and development strategy optimization.

## Discussion

The results of this study demonstrate a compelling and systematic relationship between sedimentary facies, diagenetic overprint, and the resulting hydraulic character of the reservoir, as encapsulated by the defined Hydraulic Flow Units (HFUs). This section synthesizes these findings to discuss the fundamental controls on reservoir heterogeneity and quality in the El Wastani Formation.

### Depositional environment as the primary control on reservoir architecture

The strong correlation between high-quality HFUs (HFU-4, HFU-5, HFU-6) and the high-energy Facies Sm/Sp is not coincidental but causal. These facies, interpreted as channel-fill deposits, are characterized by coarse grain size, moderate to good sorting, and a general lack of primary depositional matrix. These textural attributes create an initial pore system with large pore volumes and well-connected, large-diameter pore throats. The Kozeny-Carman relationship, which forms the basis of the FZI methodology, directly links these textural parameters to high permeability for a given porosity. Consequently, the depositional system laid the foundation for the superior mean RQI values (8.287 to 63.294 µm) observed in these units.

Conversely, the lower-quality HFUs (HFU-1, HFU-2) are consistently associated with Facies Mp and Facies Sa/Sas. These facies represent lower-energy, abandonment, or unstable depositional settings. Facies Mp, with its high mud matrix content, inherently possesses a poorly connected micro-porous system from the outset. Facies Sa/Sas, affected by slumping and sediment remobilization, exhibits poor sorting and significant argillaceous content, which occludes the primary pore network. The initial depositional porosity and permeability of these facies were low, resulting in the low mean RQI values (0.335—2.001 µm) that define HFU-1 and HFU-2.

### The role of diagenesis and clay mineralogy in pore system modification

While deposition set the template, diagenesis, particularly clay mineral authigenesis, acted as a critical modifying agent. The prevalence of mixed-layer illite/smectite (I/S) and illite, as identified by XRD and SEM, has a profound impact. The distribution of these clays is itself facies controlled. In the clean, permeable channel sands (Facies Sm/Sp), pore fluids could circulate more freely, potentially facilitating the growth of authigenic clays. However, the large pore throats in these facies can better accommodate such pore-filling minerals without a catastrophic reduction in permeability, allowing them to retain their status as high-quality HFUs.

The Thomas-Stieber model confirmed that the dominant clay distribution mode is laminated. These continuous shale lamellae act as vertical barriers or baffles to fluid flow, introducing significant small-scale anisotropy. This explains why even intervals with good reservoir rock (high FZI) can exhibit compartmentalized behavior. The laminations, a primary depositional feature, are thus a first-order control on the vertical hydraulic connectivity within the reservoir, a factor captured by the SML analysis.

### Synthesis: a genetically-linked rock typing scheme

The integration of sedimentology and petrophysics allows for the development of a genetically linked rock typing scheme that is both descriptive and predictive. The six HFUs do not merely represent arbitrary statistical clusters; they represent distinct rock types born from specific combinations of depositional process and diagenetic history.

- HFU-1 & HFU-2 are the product of low-energy, clay-rich depositional settings where the pore system is dominated by microporosity and is further compromised by diagenetic clays.
- HFU-3 likely represents a transition zone, perhaps comprising well-sorted but finer-grained sands or cleaner sands with a significant dispersed clay component.
- HFU-4 to HFU-6 are the unambiguous reservoir "sweet spots," originating from high-energy channel deposits that developed a macropore-dominated system with excellent pore connectivity.

The clear superiority of the RQI/FZI method over the Pittman R35 technique, as evidenced by the higher coefficients of determination (R^2^) for the porosity–permeability transforms (Table 3 vs. Table 4), underscores the importance of a hydraulic-based approach. The Pittman R35 method, which is based on pore-throat radii from capillary pressure, is more of a geometrical classification. The FZI method, by incorporating the tortuosity and surface area factors inherent in the Kozeny-Carman model, more effectively captures the complex interplay between depositional texture and diagenetic modification that truly governs fluid flow.

In conclusion, the reservoir quality of the El Wastani Formation is not random but is systematically governed by the interplay of depositional environment and its diagenetic sequel. The high-quality, gas-bearing sands are preferentially located within the high-energy channel facies (Sm/Sp), which possess an initial textural superiority that is largely preserved. This understanding provides a powerful predictive model: targeting the geophysical signatures of these specific depositional elements across the field will directly lead to the identification of the most productive hydraulic units, thereby de-risking future well placement and optimizing reservoir development.

## Conclusion

This study successfully implemented a novel, multi-disciplinary workflow for the comprehensive characterization of the gas-bearing El Wastani Formation in the Simian Field, offshore Nile Delta. By integrating advanced petrophysical analysis, core-based sedimentology, and hydraulic rock typing, this research has bridged a critical knowledge gap in predicting reservoir quality within this complex, clay-rich clastic system.

The key findings and contributions of this work are summarized as follows:

1. Advanced lithological and mineralogical characterization: The integration of multi-parameter cross-plots (Neutron-Density, M–N, Th-K) with core-calibrated XRD and SEM analysis definitively identified the reservoir as a sand-shale sequence, with a clay mineral assemblage dominated by mixed-layer illite/smectite. The application of the Thomas-Stieber model established laminated shale as the dominant distribution mode, a critical factor for accurately modeling resistivity and fluid saturation in shaly sands.
2. Precision in fluid properties and contacts: The free water level was unequivocally established at 2146 m TVDSS through the integration of MDT pressure gradient analysis and log responses. The formation water resistivity (Rw) was robustly constrained to 0.016 Ω·m through the exceptional concordance between direct MDT fluid sampling and Pickett plot analysis, providing a high-confidence foundation for all saturation calculations.
3. Reservoir rock typing and zonation: The core-based Flow Zone Indicator (FZI) methodology effectively classified the reservoir into six distinct Hydraulic Flow Units (HFUs 1–6), each characterized by a unique pore-throat geometry and a statistically robust, unit-specific porosity–permeability transform (R^2^ up to 0.98). This hydraulic zonation was validated by the Stratified Modified Lorenz (SML) plot, which confirmed the subdivision into six units with distinct flow-storage characteristics.
4. Depositional control on reservoir quality: The correlation between sedimentary facies and HFUs demonstrates fundamental depositional control on reservoir architecture. High-energy channel-fill deposits (Facies Sm/Sp) correspond to the highest-quality flow units (HFU-4 and HFU-5), while lower-energy facies (Mp, Sa/Sas) align with lower-quality units (HFU-1, HFU-2). This provides a powerful predictive tool for extrapolating reservoir performance across the field.
5. Superiority of integrated methodology: The comparative analysis revealed the clear superiority of the RQI/FZI approach over the Pittman R35 technique for permeability prediction in this geological setting. The RQI-derived transforms demonstrated consistently higher predictive accuracy, establishing them as the recommended method for building reliable reservoir models.
6. Pathway for field-wide application: The robust, core-defined HFU scheme established in this study provides the essential foundation for predicting reservoir quality in uncored wells and across the entire field. The logical and recommended future work is the application of machine learning techniques, such as supervised neural networks, to predict these HFUs in the Simian-2, Simian-3, and Simian-Dr wells using conventional log data. This would enable the population of a 3D reservoir model with high-fidelity, hydraulically based rock types and permeability distributions, directly leveraging the workflow and results presented in this study.

In conclusion, this integrated framework has successfully moved beyond conventional petrophysics to deliver a genetically linked rock typing scheme that captures the essential interplay between depositional environment, diagenesis, and pore-system properties. The resulting model not only enhances the understanding of the Simian Field’s reservoir heterogeneity but also provides a scalable and transferable methodology for optimizing development strategies in analogous complex clastic reservoirs within the Nile Delta and beyond. The definitive identification of high-quality, gas-saturated hydraulic units (HFUs 3–6) within specific sedimentary facies enables precise targeting of development wells and provides a robust foundation for forecasting production behavior and ultimately maximizing recovery.

## Data Availability

Data sets generated during the current study are available from the corresponding author on reasonable request, but restrictions apply to the availability of these data.
